# Impact of conditional and unconditional cash transfers on health outcomes and use of health services in humanitarian settings: a mixed-methods systematic review

**DOI:** 10.1136/bmjgh-2021-007902

**Published:** 2022-01-25

**Authors:** Kim Robin van Daalen, Sara Dada, Rosemary James, Henry Charles Ashworth, Parnian Khorsand, Jiewon Lim, Ciaran Mooney, Yasmeen Khankan, Mohammad Yasir Essar, Isla Kuhn, Helene Juillard, Karl Blanchet

**Affiliations:** 1 Cardiovascular Epidemiology Unit, Department of Public Health & Primary Care, Cambridge University, Cambridge, UK; 2 UCD Centre for Interdisciplinary Research, Education and Innovation in Health Systems, School of Nursing, Midwifery and Health Systems, University College Dublin, Dublin, Ireland; 3 University Hospitals of North Midlands NHS Trust, Stoke-on-Trent, Stoke-on-Trent, UK; 4 Harvard Medical School, Boston, Massachusetts, USA; 5 Women in Global Health, Washington, District of Columbia, USA; 6 School of Medicine, NUI Galway, Galway, Ireland; 7 Northern Ireland Medical and Dental Training Agency, Belfast, Antrim, UK; 8 Department of Biology, Siena Heights University, Adrian, Michigan, USA; 9 Kabul University of Medical Sciences, Kabul, Afghanistan; 10 Medical Library, School of Clinical Medicine, University of Cambridge, Cambridge, Cambridgeshire, UK; 11 Geneva Centre of Humanitarian Studies, University of Geneva, Geneva, Geneva, Switzerland; 12 Global Health Development, University of Geneva Faculty of Medicine, Geneve, Switzerland

**Keywords:** child health, mental health & psychiatry, nutrition, public health, systematic review

## Abstract

**Background:**

Cash transfers, payments provided by formal or informal institutions to recipients, are increasingly used in emergencies. While increasing autonomy and being supportive of local economies, cash transfers are a cost-effective method in some settings to cover basic needs and extend benefits of limited humanitarian aid budgets. Yet, the extent to which cash transfers impact health in humanitarian settings remains largely unexplored. This systematic review evaluates the evidence on the effect of cash transfers on health outcomes and health service utilisation in humanitarian contexts.

**Methods:**

Studies eligible for inclusion were peer reviewed (quantitative, qualitative and mixed-methods). Nine databases (PubMed, EMBAS, Medline, CINAHL, Global Health, Scopus, Web of Science Core Collection, SciELO and LiLACS) were searched without language and without a lower bound time restriction through 24 February 2021. The search was updated to include articles published through 8 December 2021. Data were extracted using a piloted extraction tool and quality was assessed using The Joanna Briggs Critical Appraisal Tool. Due to heterogeneity in study designs and outcomes, results were synthesised narratively and no meta-analysis was performed.

**Results:**

30 673 records were identified. After removing duplicates, 17 715 were double screened by abstract and title, and 201 in full text. Twenty-three articles from 16 countries were included reporting on nutrition outcomes, psychosocial and mental health, general/subjective health and well-being, acute illness (eg, diarrhoea, respiratory infection), diabetes control (eg, blood glucose self-monitoring, haemoglobin A1C levels) and gender-based violence. Nineteen studies reported some positive impacts on various health outcomes and use of health services, 11 reported no statistically significant impact on outcomes assessed and 4 reported potential negative impacts on health outcomes.

**Discussion:**

Although there is evidence to suggest a positive relationship between cash transfers and health outcomes in humanitarian settings, high-quality empirical evidence, that is methodologically robust, investigates a range of humanitarian settings and is conducted over longer time periods is needed. This should consider factors influencing programme implementation and the differential impact of cash transfers designed to improve health versus multipurpose cash transfers.

**PROSPERO registration number:**

CRD42021237275.

**Key questions**

**What is already known?**

- Previous studies have demonstrated the benefits of cash transfer interventions in low and middle-income countries on mitigating the health impacts from climate change, improving nutrition and advancing maternal health when markets are functional and quality services are available.
- Cash and voucher assistance amount to over US$6 billion in humanitarian aid, with cash transfers accounting for almost three-quarters of this aid.
- However, the extent to which cash transfers impact health in humanitarian settings remains largely unexplored.

**What are the new findings?**

- To our knowledge, this is the first mixed-methods systematic review exploring the impact of conditional and unconditional cash transfers specifically on health outcomes and usage of health services in a humanitarian setting.
- Health outcomes assessed in studies largely focused on diet and nutrition, mental and psychosocial health and self-reported general well-being.
- Nineteen studies reported some positive impacts on various health outcomes and use of health services, eleven reported no statistically significant impact on outcomes assessed and four reported potential negative impacts on health outcomes.

**Key questions**

**What do the new findings imply?**

- Although our systematic review suggests that there may be a positive impact of cash transfers on health outcomes in humanitarian settings, high-quality empirical evidence, that is methodologically robust, investigates a range of humanitarian settings, and is conducted over longer time periods is needed.
- Specific attention must be given to the intended/expressed purpose of grants, the actors involved in designing and implementing cash transfers, and the factors that affect implementation such as local involvement and context-specific considerations.

## Introduction

A record 274 million people are expected to need humanitarian assistance in the year of 2022,^1^ due to increasing extreme weather events, conflict and pandemics. People affected by humanitarian crises have higher rates of poor health outcomes due to increased vulnerability to violence, infectious diseases, food insecurity and chronic diseases. Collectively this picture calls for impactful, innovative solutions able to address a complex range of health challenges including both communicable and non-communicable disease prevention and treatment, water and sanitation, nutrition, access to sexual and reproductive health services and mental health and psychosocial support.^2 3^ One possible intervention to improve outcomes across these health challenges is the provision of cash transfers.

Cash and voucher assistance account for 19% of international humanitarian aid, amounting to over US$6 billion in 2020.^4^ Cash transfers compose 71% of this aid and are an increasingly common method for providing direct assistance to populations in humanitarian settings.^1 4^ Cash transfers, which are currently being used in COVID-19 pandemic responses across different settings as well as previously being provided during the Ebola epidemics,^5^ work by providing individuals with cash to access services or goods. In contrast to vouchers and in-kind assistance, cash transfers are by definition unrestricted in usage and provide recipients with physical currency or e-cash to spend. These can be conditional, where there is a prerequisite activity or obligation that the recipient must fulfil in order to receive assistance, or unconditional, where transfers are provided without the recipient having to do anything to receive the assistance.^6^

Previous studies and systematic reviews have demonstrated the benefits of cash interventions on mitigating the health impacts from climate change, improving nutrition and advancing maternal health if markets are functional and quality services are available.^7–9^ Likewise cash transfers can improve outcomes by increasing consumption of nutritious foods as well as access to preventative care and vaccinations.^10–12^ The effect of cash transfers on health systems is also thought to be beneficial by increasing incentives to seek care or lessening financial barriers to access care.^10^ Additionally, cash transfers are often preferred by recipients and can offer certain benefits over the direct provision of food, goods or services such as cost-effectiveness, rapid and flexible implementation and transparency.^13 14^ Cash may also empower recipients by providing autonomy to spend money based on personal need and improve economic growth and stability by enabling the purchase of goods and services freely on the local markets.^15 16^ While cash transfers offer this flexibility, their effectiveness is also influenced by when and how they are provided (eg, delivery approaches).^17^ How these interventions are designed and implemented influences the impact of the aid; some of these implementation factors include who is targeted as recipients and their value for money, the state of local markets and infrastructure, available resources, community acceptance and risks relating to security in the setting.^14 18 19^

To date, multiple systematic reviews have explored the impact of cash transfers on human health or well-being.^10 14 19–24^ The findings of these reviews have been relatively consistent, suggesting the beneficial impact of cash transfers on the health outcomes of individuals and communities. For example, reviews have identified the utility of conditional cash transfer programmes in improving access to preventive services, while also acknowledging the potential influences of other components.^10 20^ However, it is important to consider the unique contexts and challenges of populations in humanitarian crises over the general population. Humanitarian settings may be characterised by disrupted health systems and supply chains as well as political and economic instability. People living in humanitarian settings are, therefore, more vulnerable to illness, and subject to worse access to health services and care, and health protection, than the average population. These are contextual factors that need to be considered in the delivery of any health intervention that may not necessarily be addressed in a more static, stable community. Only one systematic review^22^ specifically examined the effect on health outcomes and the utilisation of health services in humanitarian settings. This review, conducted 6 years ago, focuses solely on unconditional cash transfers (UCTs) and only synthesised three low-quality studies focused on drought contexts. Therefore, in this, we assess the impact of conditional and UCTs on health outcomes and utilisation of health services in humanitarian settings to provide evidence informing future cash transfer interventions and humanitarian response.

## Methodos

This systematic review protocol was prospectively registered on PROSPERO (CRD42021237275). Findings were reported following the Preferred Reporting Items for Systematic Reviews and Meta-Analyses guidelines (online supplemental table 7).^7^

10.1136/bmjgh-2021-007902.supp1 Supplementary data

### Definitions

Cash transfers were defined following The Cash Learning Partnership definition as cash payments (physical currency or e-cash) provided by formal and/or (eg, government, non-governmental organisations) informal (eg, hawala—an informal money transfer system largely used in the Middle East and South Asia)^25^ institutions to recipients that enable them to meet minimum life necessities.^6^ Unconditional (transfers are provided without the recipient having to do anything to receive the assistance), conditional (there is a prerequisite activity or obligation that the recipient must fulfil in order to receive assistance, eg, attendance of an educational course) and short-duration and ongoing cash transfer interventions were included. Cash transfers are unrestricted, in contrast with (food) vouchers and in-kind assistance.^6^

The primary outcome(s) were changed in health outcomes, including mortality, morbidity, (mal)nutrition, mental health and well-being and acute or chronic disease status. Secondary outcome(s) were the utilisation of health services, measured by the frequency of visits or percentage of population eligible for a service attending the service.

A humanitarian setting was defined as an event or series of events that present a critical threat to the health, safety, security or well-being of a community or other large group of people, usually over a wide geographic area. Three types of humanitarian crises were specified: man-made crises (eg, civil and inter-state war, armed conflict, genocide), natural disasters including hydrological (eg, floods, avalanches), geophysical (eg, earthquakes, volcanic eruptions, earthquakes), climatological (eg, droughts, wildfires), meteorological (eg, storms, cyclones), biological events (eg, pandemics, epidemics, plagues) and complex emergencies (emergencies resulting from a combination of both natural and man-made causes).^26–28^

### Search methods and information sources

We searched nine electronic databases (PubMed, EMBASE via Ovid, Medline via Ovid, CINAHL via EbscoHost, Global Health via EbscoHost, Scopus, Web of Science Core Collection, SciELO and LiLACS) without restriction of language and without a lower bound time restriction for articles published through 24 February 2021. An updated search was conducted to include articles published during the COVID-19 crisis through 8 December 2021. Using a combination of free-text terms and subject headings, we used vocabulary related to ‘cash transfers’ and ‘humanitarian settings’. The full-search strategy, developed with a librarian/information specialist, is provided in online supplemental table 1. We conducted forward and backward screening of all articles in the full-text screening phase as well as relevant publications (eg, reviews, opinion pieces) to find any additional studies fitting the inclusion criteria. We also searched Google Scholar to find additional publications.

### Study selection

Eight researchers were involved in the study selection and extraction. After removing duplicates using Endnote, abstracts and titles were screened independently by two researchers according to the selection criteria by using the software Rayyan (https://rayyan.ai/). Studies satisfying the inclusion criteria were retrieved and screened by full text. Conflicts between two authors screening the same studies were resolved among authors until consensus was reached at both stages. A third arbiter was involved when consensus could not be reached. We included primary peer-reviewed quantitative, qualitative and mixed-method studies that either (1) reported on the effect of cash transfers on health outcomes in humanitarian settings or (2) reported on the effect of cash transfers on healthcare utilisation in humanitarian settings. We excluded studies that were (1) non-human studies, (2) conference proceedings or secondary studies (eg, reviews), (3) lacking a full text. In order to capture rigorous data of peer-reviewed studies and evidence, we did not include grey literature (such as non-governmental organisation (NGO) reports). As our research team is fluent in a range of languages (including Arabic, Dutch, English, Farsi, French, German, Pashto, Persian, Spanish, Urdu), we did not exclude any articles based on language; non-English full texts were translated or reviewed by a native or fluent speaker.

### Data extraction and study quality assessment

Data from included studies were independently extracted in duplo using a pretested extraction tool. Any discrepancies between authors extracting the same studies were discussed until consensus was reached. Likewise, a third arbiter was involved when consensus could not be reached. The following information was extracted for each study: author, year, study title, study design, study population, participant demographics (eg, age, country), type of humanitarian setting, sampling and recruitment procedures, total number of participants, outcome (use of health services, health outcomes), outcome(s) ascertainment, type of cash transfer, percentage/number of individuals reporting the outcome, association measures with summary estimate and 95% CI. An open field to record any additional relevant information was available. The quality of individual included studies was assessed using the Joanna Briggs Institute (JBI) critical appraisal tool to explore methodological quality of the synthesised knowledge.^8^

### Statistical and thematic analysis

Due to the heterogeneity of the included studies (in type of cash transfer, outcome and setting), the quantitative data were descriptively synthesised, and no meta-analysis was performed. Studies with qualitative data underwent additional qualitative thematic analysis. Authors independently used inductive analysis to develop and agree on a codebook. This codebook was then applied to all qualitative studies by two independent authors (inter-rater reliability kappa score 0.96).

### Patient and public involvement

Due to the nature of this study (systematic review), no patients or public were involved in conceptualising or conducting the study.

## Results

### Characteristics of included publications

**Figure 1:**
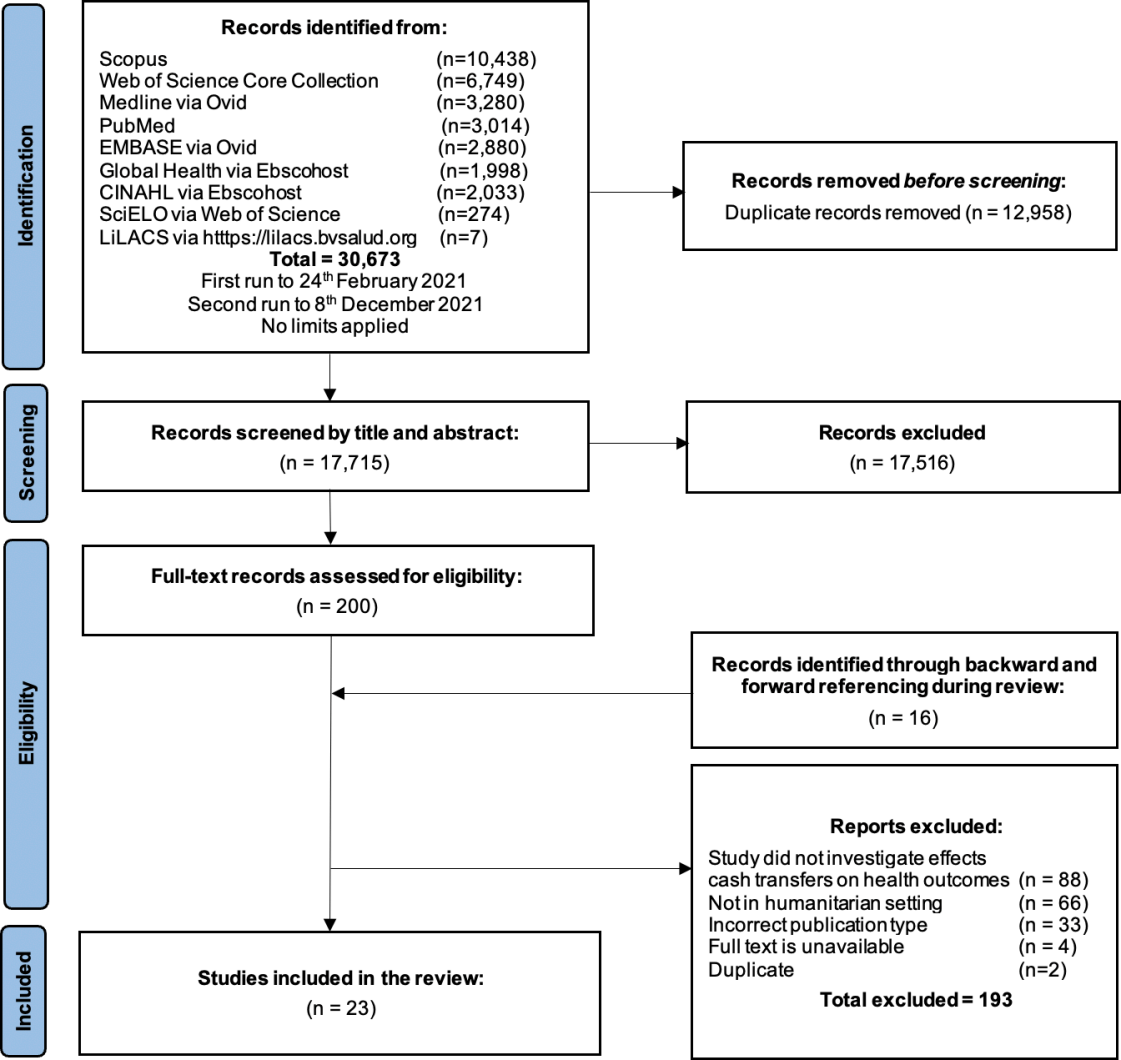
Flow diagram of included studies.

We identified 30 673 records from the databases. After removing duplicates, 17 715 records were screened by title and abstract and 201 were screened in full text (figure 1). In total, 23 articles were included in this review and their summary characteristics are reported in table 1.^29–51^Table 2 provides summaries of the main results of the included studies. Included studies were conducted in 16 countries: Niger (n=3),^31 32 46^ Somalia (n=3),^33 34 39^ Afghanistan (n=2),^35 37^ Jordan (n=2),^41 48^ Lebanon (n=2),^49 50^ Yemen (n=2),^45 47^ Palestine,^29^ Democratic Republic of Congo,^30^ Syria,^36^ Cameroon,^37^ Uganda,^38^ Bangladesh,^40^ Ecuador,^42^ Mexico,^43^ Kenya^44^ and Togo.^51^ Eighteen of the studies were quantitative,^30–35 38 39 42–51^ two studies were qualitative^37 41^ and three studies were mixed methods.^29 36 40^ This included several study designs: randomised control trials (n=7),^30 38 42 43 45–47^ cohort studies (n=5),^31 33 34 36 49^ quasi-experimental studies (n=6),^29 32 40 44 48 50^ non-randomised control trials (n=2),^35 39^ a cross-sectional study^51^ and qualitative study designs using in-depth interviews (n=5),^29 36 37 40 41^ focus group discussions (n=4)^29 37 40 41^ or observations (n=1).^29^ The number of participants included in studies ranged from 140 individuals^41^ to approximately 24 000 households.^43^ Though no time restriction was applied to the search, all studies were published after 2010, with the vast majority published since 2018.^32–37 39–41 44 45 47^ Studies were conducted as early as 1998–2000 43 and as recently as July 2020 (during the COVID-19 pandemic).^51^

**Table 1:** Summary characteristics of included studies

Study	Study design	Study period	Country	Type of humanitarian setting	Type of cash transfer (intervention)	Health outcome/services	Population source	N participants
Abu-Hamad *et al.* 2014^29^	Mixed-methods *Quantitative:* Quasi-experimental *Qualitative:* In-depth interviews, FGDs, key informant interviews, observations	April–May 2013 June/July 2013	Gaza, Palestine	Man-made crises (human conflict)	Unconditional cash transfer Monetary value and time-period not given.	-Psychological health	Children (<18 years) benefiting from the Palestinian National Cash Transfer Programme (PNCTP) and caregivers living in Gaza Key informants (eg, gov officials, NGOs) were also interviewed	Quantitative: 4497 people Qualitative: Small group discussion: 74 children In-depth interviews: 10 children Observations: 2 HH FGD: 14 adults Key informant interviews: 11
Aker *et al.* 2017^30^	Randomised control trial	August/September 2011–March 2012	Democratic Republic of the Congo	Man-made crisis (human conflict)	Unconditional cash transfer US$130 over 7-month period in three disbursements (September, November, February 2012)	Household member affected by illness or died Expenditure on medicine	Informal camp in the Masisi territory of DRC, total population ~2500 individuals.	474 households (237 cash intervention, 237 comparison group receiving vouchers)
Bliss *et al.* 2016^31^	Longitudinal cohort study	April–September 2012	Niger	Environmental (food crisis)	Unconditional cash transfer US$296 over 6-month period in monthly disbursements. (160 000 West African Francs)	Presence of acute malnutrition (WHZ <2 or MUAC <125 mm) MUAC WHZ Child dietary diversity Child meal frequency Child illness Maternal mental health	Children aged 6–36 months in 420 households enrolled in an emergency cash transfer programme in Niger	420 households (all enrolled in the intervention)
Bliss *et al.* 2018^32^	Quasi-experimental	July–September 2012	Niger	Environmental (food crisis)	Conditional cash transfer *condition:* Mothers to attend a health and nutrition education session prior to each cash transfer ~US$250 over 3-month period in 3 monthly disbursements (125 000 West African Francs)	Weight gain Weight gain velocity (g/kg/d) MUAC WHZ Presence of acute malnutrition (WHZ <2 or MUAC <125 mm or the presence of bilateral pitting oedema) Meal frequency Dietary diversity	Households in a conditional emergency CTP programme that occupied the second-lowest wealth category and had a child 6–23 months that was not wasted or had diseases.	423 households (211 cash intervention, 212 comparison group that did not receive cash)
Doocy *et al.* 2020 A^33^	Prospective cohort study	November 2018–April 2019	Somalia	Man-made crises (human conflict) Environmental (drought, food crisis)	Unconditional cash transfer combined with in-kind food and electronic vouchers. ~US$450 over 4-month period	Household Hunger Scale (HHS) Minimum Dietary Diversity for Women (MDDW) MUAC, with MUAC <21.0 cm classified as acute malnutrition Meal frequency	Pregnant and lactating women in El-bon Camp in the District of Wajid and adjacent neighbourhoods for 'non-assistance' group.	514 pregnant and lactating women (baseline and endline comparison)
Doocy *et al.* 2020 B 34	Prospective cohort study	November 2018–April 2019	Somalia	Man-made crises (human conflict) Environmental (drought, food crisis)	Unconditional cash transfer combined with in-kind food and electronic vouchers. ~US$450 over 4-month period	Household Hunger Scale (HHS) Children’s dietary diversity Meal frequency Minimum acceptable diet (MAD) Mean MUAC Acute malnutrition (defined as MUAC <12.5 cm)	Households in El-bon Camp in the District of Wajid and adjacent neighbourhoods for 'non-assistance' group with children aged 6–59 months.	490 households (n=269 mixed transfer group (cash, in-kind, voucher), n=162 food voucher group comparison group, n=59 no assistance comparison group)
Edmond *et al.* 2019^35^	Non-randomised population-based intervention study	December 2016–December 2017	Afghanistan	Man-made crises (human conflict)	Conditional cash transfer *condition:* for mother delivery at a health facility and for CHW when accompanying/referring a mother to the health facility ~US$15 one-time (mother) (1000 Afghani),~US$5 one-time (CHW) (300 Afghani)	Child delivery in a health facility Receiving at least one ANC visit Receiving one PNC visit Receiving at least one CHW home visit	Women that had given birth to one or more children in the last 12 months residing in the study districts of the three provinces (Badghis, Bamyan and Kandahar)	4929 women (2453 intervention with 1199 baseline, 1254 endline) (2476 control receiving standard care with 1242 baseline, 1234 endline)
Falb *et al.* 2020^36^	Mixed methods *Quantitative:* Prospective cohort study *Qualitative:* in-depth interviews	March–August 2018	Syria	Man-made crises (human conflict)	Unconditional cash transfer US$228 over a 3-month period in monthly disbursements.	Food insecurity via HFIAS Depressive via PHQ-9 Disability status, via an adapted version of the WG-SS Women’s experiences of violence	All HH with a woman aged 18–59 years from a beneficiary household in Raqqa Governorate, Syria.	512 women at baseline, 456 at endline (baseline and endline comparison)
Freccero *et al.* 2019^37^	Qualitative study (focus groups+in-depth interviews)	2017–2018	Cameroon, Afghanistan	Man-made crises (human conflict)	Cameroon: multipurpose cash programme (~US$300 over a 6-month period in monthly disbursements) Afghanistan: one-time amount for non-food items (~US$80–198) (for both: unclear whether conditional or unconditional)	Self-reported changes in health (not more detail provided on specific health outcomes)	Participants receiving multipurpose cash transfers through International Red Cross programmes in Cameroon and Afghanistan.	211 individuals, 100 Afghanistan, 111 Cameroon (all enrolled in intervention)
Green *et al.* 2016^38^	Cluster-randomised trial	April 2009	Uganda	Man-made crises (human conflict)	Unconditional cash transfer ~US$150 one-time combined with a programme for business skills training and follow-up support	Depression using modified version APAI depression subscale	120 communities (villages, transit sites, and displacement camps in Gulu and Kitgum districts) in Northern Uganda	1800 individuals (868 intervention receiving cash, 866 comparison group not receiving cash)
Grijalva-Eternod *et al.* 2018^39^	Non-randomised cluster trial	March–November 2016	Somalia	Man-made crises (IDP camps)	Unconditional cash transfer ~US$420 over a 5- month period in monthly disbursements	Mean child, household and women DDS values Incidence acute malnutrition, defined by low MUAC or oedema Prevalence acute malnutrition, defined by low WHZ or oedema Mean WHZ value Mean FCS Mean HFIAS score Mean Reduced Coping Strategies Index (rCSI) score	IDP camps located in Weydow area, Deyniile district, Mogadishu. Recipients of cash transfer were female household representatives	228 households (111 intervention, 117 comparison not receiving cash) with 332 children (155 intervention, 177 comparison not receiving cash)
Gros *et al.* 2019^40^	Mixed-methods *Quantitative:* quasi-experimental *Qualitative*: focus groups and in-depth interviews	May 2016–October 2017	Bangladesh	Environmental (flooding)	Unconditional cash transfer (forecast-based) ~US$60 one-time (5000 Bangladesh taka)	Psychosocial distress Health expenses	Poor households in flood-prone communities of the Brahmaputra river basin.	390 households (174 intervention, 216 comparison not receiving cash)
Hagen-Zanker *et al.* 2018^41^	Qualitative study (focus groups+in-depth semi-structured interviews)	2016	Jordan	Man-made crises (refugees)	Unconditional cash transfer Monetary value and time-period not given.	Self-reported effect on access to health, stress, and anxiety	Working age Syrian refugees in Jordan and key informants (policy-makers, practitioners at national level)	Over 140 Syrian refugees across 60 interviews and FGDs (37 interviews and 7 FGDs intervention, 10 interviews and 5 FGDs comparison group not receiving cash)
Hidrobo *et al.* 2014^42^	Randomised control trial	May–October 2011	Ecuador	Man-made crises (IDP camps)	Unconditional cash transfer US$240 over 6-month period in 6 monthly disbursements	DDS HDDS FCS Caloric intake per capita (daily)	Colombian refugees and Ecuadorian locals in seven urban centres in the provinces of Carchi and Sucumbíos	2087 households receiving either cash, food or control (unclear N for each group)
Hou 2010^43^	Randomised control trial	1998–2000	Mexico	Environmental (drought)	Conditional cash transfer *condition:* not given Monetary value and time-period not given.	Total calorie consumption Diversity of diet including vegetables, fruits and animal products	HH across seven states in Guerrero, Hidalgo, Michoacan, Puebla, Queretaro, San Luis Potosi, and Veracruz	10 541 (6362 intervention, 4179 controls not receiving cash)
Kurdi 2021^47^	Cluster randomised control trial	Baseline sample: December 2014–January 2015 Follow-up sample: July–August 2017	Yemen	Man-made crisis (Civil war)	Conditional cash transfer *condition:* attendance of nutritional training and compliance with monitoring and treatment of malnutrition 2015:~42 US$ per woman per 3 months in 2015 (9000 Yemeni riyal) 2016/2017:~28 US$ per woman in 10–11 monthly disbursements	HDDS CDDS Height-for-age	HH with young children in Yemen. To meet treatment arm criteria women had to be “second priority” potential beneficiaries.	Baseline: 2000 HH (1001 treatment, 999 control HH) Follow-up: 1850 re-surveyed, 95 additional replacement HH
Lyles *et al.* 2021 A^48^	Quasi-experimental prospective cohort	October 2018–January 2020	Jordan	Man-made crisis (refugees)	CHV intervention +conditional cash transfer group *condition:* quarterly group education sessions and home visits, usage of appropriate services requiring participants to provide receipts ~211 US$ per person per 3 months (150 Jordanian Dinar) Multi-purpose unconditional cash (MPC) transfer group ~113–219 US$ per household per 3 months (80–155 Jordanian Dinar) Time period not given	Health service utilisation (diabetes care visits) Diabetes medication adherence Blood glucose self-monitoring BMI HbA1C Blood pressure Health expenditure	Syrian refugees with type II diabetes residing outside of camps in Amman and Zarqa governorates of Jordan	Baseline: 560 (156 CHV only, 203, CHV +CCT, 201 MPC) Endline: 482 (128 CHV only, 179 CHV +CCT, 175 MPC)
Lyles *et al.* 2021 B^49^	Prospective cohort	May 2018–July 2019	Lebanon	Man-made crisis (refugees)	Multi-purpose unconditional cash transfer ~173.5 US$ per family per month (260 000 Lebanese pound) Time period not given.	Health service utilisation (care-seeking for children and chronic or acute illness among adults) Access to medication Health expenditure	Vulnerable Syrian refugee households sampled from UNHCR registration lists receiving MPCs (intervention) and similarly vulnerable households not receiving MPCs	Baseline: 617 HH (173 MPC intervention, 444 control group) Endline: 168 MPC, 375 control group (follow-up 1 year)
MacPherson and Sterck 2021^44^	Quasi-experimental	September–October 2017	Kenya	Man-made crises (refugee settlement)	Cash transfer (unclear whether conditional or unconditional) ~US$14–17 per person per month (unclear time period) (1400–1708 Kenyan Shilling)	DDS Calories per adult equivalent HFIAS Subjective well-being	Refugees in Kakuma camp and Kalobeyei settlement in Kenya	1874 refugees (1126 households) (914 intervention, 960 control (Kakuma camp))
Moussa *et al.* 2022^50^	Quasi-experimental (regression discontinuity design)	Survey (wave 1): February–March 2019 Survey (wave 2): July–August 2019	Lebanon	Man-made crises (refugees)	Multi-purpose unconditional cash transfer (monetary value unclear) Discontinued (12 months) Short run (up to 10 months) Long-term (16–22 months)	Acute illness Diarrhoea Respiratory infection Needed primary healthcare Accessed primary healthcare	Syrian refugee children (<19 years) from discontinued cash recipient households, short-run and long-term recipient households living in Lebanon	6,207 HH (2992 wave 1, 3215 wave 2) with 24 859 observations (11 843 wave 1, 13 016 wave 2)
Schwab 2020^45^	Cluster randomised control trial	November 2011–October 2012	Yemen	Man-made crises (human conflict)	Unconditional cash transfer ~US$147 over 3-month period in 3 monthly disbursements (10 500 Yemeni riyals)	HDSS FCS Value of food consumed Caloric intake	135 village clusters in rural Yemen.	1983 people (982 intervention receiving cash, 1001 comparison receiving in-kind food)
Sibson *et al.* 2018^46^	Cluster randomised control trial	March 2015–November 2015	Niger	Environmental (food-crisis)	Unconditional cash transfer Standard intervention = ~£144 over 4 month period in 4 monthly disbursements Modified intervention = ~ £144 over 6 month period in 6 monthly disbursements	Acute Malnutrition MUAC WHZ Dietary diversity	Children aged 6–59 months, living in villages receiving unconditional cash transfer.	1130 HH standard intervention 963 HH modified intervention. Sampled 1959 children and obtained baseline measures from 1831 (2093 intervention, 495 control)
Tossou 2021^51^	Cross-sectional	July 2020	Togo	Environmental (COVID-19 pandemic)	Unconditional cash transfer Monetary value and time period not given.	Health service and healthcare utilisation	National household survey covering 44 districts in six health regions in Togo: HH heads, consenting adults, children 10–17 years were surveyed	955 beneficiaries

ANC, antenatal care; APAI, Acholi Psychosocial Assessment Instrument; CHV, community health volunteer; CHW, community healthcare worker; CTP, cash transfer program.programme; DDS, dietary diversity score; DRC, Democratic Republic of the Congo; FCS, food consumption score; HFIAS, household food insecurity access scale; HH, household; HHS, household hunger scale; IDP, internally displaced people; MAD, minimum acceptable diet; MDDW, minimum dietary diversity for women; MUAC, mid-upper arm circumference; PHQ-9, Patient Health Questionnaire; PNC, postnatal care; PNCTP, Palestinian National Cash Transfer Programme; WG-SS, Washington group disability short set; WHZ, waist-to-hip ratio.

**Table 2:** Summary of main results and conclusions

Study	Country	Type of humanitarian setting	Type of cash transfer	Health outcome/services	Main results and/or conclusion
Abu-Hamad *et al.* 2014^29^	Gaza, Palestine	Man-made crises (human conflict)	Unconditional cash transfer Monetary value and time period not given.	Psychological health measured by a Self-Esteem Scale containing nine questions, paying for healthcare	*Psychosocial and Mental Health measures through a self-esteem scale* A Self-Esteem Scale showed that the intervention group had a higher overall score (0.73) compared with the comparison group on the waiting list to receive cash transfers (0.68). 7.55% of children in the intervention group had an abnormal Strength and Difficulties Questionnaire (behavioural health screening tool) score compared with 9.18% in the comparison group.
Aker 2017^30^	Democratic Republic of the Congo	Man-made crisis (human conflict)	Unconditional cash transfer US$130 over 7 month period in three disbursements (September, November, February 2012)	Household member affected by illness or died Expenditure on medicine	*Prevalence of illness and death among household members* Affected by illness: −0.01 (0.08) cash, 0.59 (0.50) comparison using voucher (*P*-value 0.87) Death: 0.03 (0.05) cash, 0.11 (0.31) comparison using voucher (p value 0.57) *Health Expenditure* Households receiving cash transfers were more likely to use the funds to pay for health expenses
Bliss *et al.* 2016^31^	Niger	Environmental (food crisis)	Unconditional cash transfer US$296 over 6 month period in monthly disbursements. (160 000 West African Francs)	Presence of acute malnutrition (WHZ <2 or MUAC <125 mm) MUAC WHZ Child dietary diversity Child meal frequency Child illness Maternal mental health	*Diet and Nutrition* Factors found to be associated with risk of acute malnutrition in households receiving cash transfers included low WHZ, household poverty status, and occurrence of child illness. Household food expenditures and other diet-related factors were not found to be associated with the risk of acute malnutrition. Over the course of the study, 18% (n=74) children in the cash transfer programme became acutely malnourished.
Bliss *et al.* 2018^32^	Niger	Environmental (food crisis)	Conditional cash transfer *condition:* Mothers to attend a health and nutrition education session prior to each cash transfer ~US$250 over 3 month period in 3 monthly disbursements (July, August, September) (125 000 West African Francs)	Weight gain Weight gain velocity (g/kg/d) MUAC WHZ Presence of acute malnutrition (WHZ <2 or MUAC <125 mm or bilateral pitting oedema) Meal frequency Dietary diversity	*Diet and nutrition* *Weight Gain:* difference estimations found that cash transfers were associated with a 1.27 kg greater overall weight gain (p<0.001) compared with the control group that did not receive cash. *Weight-for-height Z scores:* 1.82 greater overall gain in WHZ (p value<0.001) in the cash group. *Acute malnutrition:* odds of acute malnutrition at the end of the intervention was 25 times higher among children in the comparison group compared those in the intervention group receiving cash (p value<0.001). *Meal frequency:* compared to the comparison group, increased by one meal more on average among cash group (p value<0.001). *Dietary diversity:* children in cash group consumed more food groups (p value<0.001).
Doocy *et al.* 2020 A^33^	Somalia	Man-made crises (human conflict) Environmental (drought, food crisis)	Unconditional cash transfer combined with in-kind food and electronic vouchers. ~US$450 over 4-month period	Household Hunger Scale (HHS) Minimum Dietary Diversity for Women (MDDW) MUAC (MUAC <21.0 cm classified as acute malnutrition) Meal frequency	*Diet and nutrition* *Hunger:* in the mixed transfer group fewer households reported moderate or severe hunger (35.4% compared with 44.0% and 94.9% in voucher and non-assistance groups) *Meal frequency: t*here was a significant increase in meal frequency of cash transfer recipients (0.3 meals/day, CI: 0.1 to 0.5, p value 0.001) compared with voucher recipients. *MUAC:* an increase in mean MUAC was statistically significant in both voucher (0.9 cm, CI: 0.6 to 1.3, p=0.001) and mixed transfer recipients (1.3 cm, CI: 1.1 to 1.5, p value 0.001). Yet, mean MUAC increased by 0.4 cm (95% CI: −0.1 to 0.8, p value 0.086) more in mixed transfer compared with voucher. *MDDW:* adjusted model reported no significant difference between mixed transfer and voucher (7.7%, 95% CI: −7.0 to 22.5, p value 0.3)
Doocy *et al.* 2020 B^34^	Somalia	Man-made crises (human conflict) Environmental (drought, food crisis)	Unconditional cash transfer combined with in-kind food and electronic vouchers. ~US$450 over 4-month period	Household Hunger Scale (HHS) Children’s dietary diversity Meal frequency Minimum acceptable diet (MAD) Mean MUAC Acute malnutrition (MUAC <12.5 cm)	*Diet and Nutrition* *Hunger:* 56.0% of the food voucher group and 64.6% of the mixed transfer group were classified as having little to no hunger at baseline *Dietary diversity:* 64.4% of children in the mixed transfer group compared with 51.0% of children in the food voucher group achieved the minimum dietary diversity *MUAC:* the mean MUAC values increased significantly by 0.4 cm (CI: 0.1 to 0.8, p=0.001) in the food voucher group, but there was no statistically significant change in the mixed transfer group (−0.1, CI: −0.4 to 0.2, p=0.001). *Acute malnutrition:* while it was not statistically significant, the prevalence of acute malnutrition was 2.9% (CI: 0.4 to 6.1, p=0.086) greater in the food voucher group as compared with the mixed transfer group.
Edmond *et al.* 2019^35^	Afghanistan	Man-made crises (human conflict)	Conditional cash transfer *condition:* for mother delivery at a health facility and for CHW when accompanying/referring a mother to the health facility ~US$15 one-time (mother) (1000 Afghani),~US$5 one-time (CHW) (300 Afghani)	Child delivery in a health facility Receiving at least one ANC visit Receiving one PNC visit Receiving at least one CHW home visit	*Health Service Utilisation* *ANC visits:* there was a statistically significant increase in ANC visits in the intervention villages (AMD 45.0%, CI: 0.18 to.0.72, p value 0.004). *Facility delivery*: facility deliveries (AMD 3.3%, CI: −0.14 to 0.21, p=0.685) and PNC visits (AMD 31.8%, CI: −0.05 to 0.68, p=0.080) also increased, but these findings were not statistically significant. *CHW Home visits:* CHW home visits did not change significantly in the intervention villages, but did decrease by 23.9% in control villages (AMD 12.2%, CI: −0.27 to 0.51, p=0.508).
Falb *et al.* 2020^36^	Syria	Man-made crises (human conflict)	Unconditional cash transfer US$228 over a 3-month period in monthly disbursements.	Food insecurity via (HFIAS) Depressive symptoms via the PHQ-9 Disability status, via an adapted version of the WG-SS Women’s experiences of violence	*Diet and Nutrition* Between baseline and endline, Food insecurity items decreased by 0.92 points (95% CI: −1.17 to −0.68; p<0.0001) in the unadjusted linear model This significant decrease in food insecurity remained robust when adjusting for demographics (β=−0.90; 95% CI: −1.14 to −0.65; p<0.0001) Or when includinghousehold fixed effects (β=−0.95; 95% CI: −1.19 to −0.71; p<0.0001) *Psychosocial and Mental Health* Women agreed on average with 12.08 statements at baseline (SD: 3.32) on the 20 item HESPER scale. This signifieshigh household daily stressors and perceived serious needs. This was similar at endline (Mean: 12.11; SD: 3.87). Unadjusted, adjusted and household fixed effects models were not statistically significant (β=0.04; p=0.83; β=0.05; p=0.81; β=0.12; p=0.52, respectively). Women had a mean of 11.08 on the PHQ-9 scale on average at baseline and 11.93 at endline. Between baseline and endline, depressive symptoms changed by 0.86 points (95% CI: 0.32 to 1.40; p=0.002) in the unadjusted model. This was similar in the adjusted model (β=0.92; 95% CI: 0.35 to 1.49; p=0.001) as well as in the household fixed effects model (β=0.89; 95% CI: 0.34 to 1.43; p=0.001).
Freccero *et al.* 2019^37^	Cameroon, Afghanistan	Man-made crises (human conflict)	Cameroon: multipurpose cash programme (~US$300 over a 6 month period in monthly disbursements) Afghanistan: one-time amount for non-food items (~US$80–198) (for both: unclear whether conditional or unconditional)	Self-reported changes in health	*General Health and Wellbeing* At the individual and household levels, many respondents reported improvements in health. No additional detail was provided.
Green *et al.* 2016^38^	Uganda	Man-made crises (human conflict)	Unconditional cash transfer ~US$150 one-time combined with a programme for business skills training and follow-up support.	Depression using modified version APAI depression subscale	*Psychosocial and Mental Health* There were decreases in depression severity in both the treatment and control groups over time. At endline, the treatment group mean decreased by 29%, from 0.85 to 0.60. Likewise, the control group mean decreased by 21%, from 0.75 to 0.59 The average treatment effect on symptoms of depression was not statistically significant and small
Grijalva-Eternod *et al.* 2018^39^	Somalia	Man-made crises (IDP camps)	Unconditional cash transfer ~US$420 over a 5 month period in monthly disbursements	Mean child, household and women DDS values Incidence acute malnutrition, defined by low MUAC or oedema Prevalence acute malnutrition, defined by low WHZ or oedema Mean WHZ value Mean FCS Mean HFIAS score Mean Reduced Coping Strategies Index (rCSI) score	*Diet and Nutrition* Cash based initiatives (CBIs): Increased Child Dietary Diversity score by 0.53 (95% CI 0.01 to 1.05) Increased monthly household expenditure by US$29.60 (95% CI 3.51 to 55.68) Increased household Food Consumption Score by 14.8 (95% CI 4,83 to 24.8) Decreased Reduced Coping Strategies Index by 11.6 (95% CI 17.5 to 5.96) Did not reduce risk of acute childhood malnutrition
Gros *et al.* 2019^40^	Bangladesh	Environmental (flooding)	Unconditional cash transfer (forecast-based) ~US$60 one-time (5000 Bangladesh taka)	Psychosocial distress Health expenses	*Diet and Nutrition* No significant difference in change in dietary quality observed between food voucher and mixed transfer recipients A significant difference in change in mean meal frequency was observed (0.3 meals/day, CI: 0.1 to 0.5, p=0.001). Mean MUAC increased significantly among both voucher (0.9 cm, CI: 0.6 to 1.3, p=0.001) and mixed transfer recipients (1.3 cm, CI: 1.1 to 1.5, p=0.001) Fewer households in the mixed transfer group had moderate or severe hunger (35.4% compared with 44.0% and 94.9% in voucher and non-assistance groups, respectively) *Psychosocial and Mental Health* After the flood, households not receiving FbF assistance felt miserable or unhappy significantly more frequently compared to the intervention group not receiving cash assistance In the last seven days before the survey, compared to the intervention group, FbF-assisted households were significantly less likely to have felt anxious or depressed.
Hagen-Zanker *et al.* 2018^41^	Jordan	Man-made crises (refugees)	Unconditional cash transfer Monetary value and time-period not given.	Self-reported effect on access to health, stress and anxiety	*Psychosocial and Mental Health* A third of participants reported the cash transfer improved their mental well-being. The cash transfers also alleviated stress or anxiety related to paying rent. *Health Expenditure* The cash transfer helped to reduce small health expenditures by enabling recipients to partially cover the costs of treatment or medication. For other beneficiaries it helped to secure a loan covering healthcare expenses. Whilst the cash transfers alleviated some financial burdens of accessing healthcare, this was not a decisive factor in recipients' behaviours related to accessing health treatment.
Hidrobo *et al.* 2014^42^	Ecuador	Man-made crises (IDP camps)	Unconditional cash transfer US$240 over 6-month period in 6 monthly disbursements	DDS HDDS FCS Caloric intake per capita (daily)	*Diet and Nutrition* All three groups (cash, food vouchers, food transfers) experienced significant improvements in households’ caloric intake and dietary diversity, however caloric intake increased by 21% in the food group and only by 12% in the cash group (p=0.05). The FCS, which measures households’ food consumption, increased by 11% in the cash group, 12% in the food group, and 16% in the voucher group. However, only the voucher and food groups saw statistically significant reductions in the percentage of households with poor to borderline FCS
Hou 2010^43^	Mexico	Environmental (drought)	Conditional cash transfer *condition:* not given Monetary value and time-period not given.	Total calorie consumption Diversity of diet including vegetables, fruits, and animal products	*Diet and Nutrition* When drought affects income, households tend to buy cheaper calories (such as grains), which results in a net increase in total calories consumed, but these calories are more likely to cause chronic diseases. The CCT (PROGRESA) mitigates the negative effects of drought on calorie availability from fruits, vegetables and other sources. The CCT does not mitigate the impact of drought on calories from grains.
Kurdi 2021^47^	Yemen	Man-made crisis (Civil war)	Conditional cash transfer *condition:* attendance of nutritional training and compliance with monitoring and treatment of malnutrition 2015:~42 US$ per woman per 3 months (9000 Yemeni riyal (YER)) 2016/2017:~28 US$ per woman in 10–11 monthly disbursements	HDDS CDDS Height-for-age z score (HAZ)	*Diet and Nutrition* Positive significant (large) impact on dietary diversity across full sample, strongest in poorest HH Overall the intervention increased the CDDS by 0.61 food groups across all HH Average estimated programme impact on HAZ across all HH was not significant HAZ of HH in lowest tercile statistically significant and large impact of 0.31 SD
Lyles *et al.* 2021 A^48^	Jordan	Man-made crisis (refugees)	CHV intervention +conditional cash transfer group *condition:* quarterly group education sessions and home visits, usage of appropriate services requiring participants to provide receipts ~211 US$ per person per 3 months (150 Jordanian Dinar) Multi-purpose unconditional cash (MPC) transfer group ~113–219 US$ per household per 3 months (80–155 Jordanian Dinar) Time period not given.	Health service utilisation Diabetes medication adherence Blood glucose self-monitoring BMI HbA1C Blood pressure Health expenditure	*Health Service Utilisation (diabetes care)* Regular diabetes care visits increased in the CHV + CCT group (15.1%, CI: 5.4,24.8%; p= 0.002) Specialist visits increased among CHV +CCT group (16.8%, CI: 6.6 to 27.0%; p= 0.001) Specialist visits decreased in the CHV only participants (− 27.8%, CI: − 41.5,% to 14.0%; p < 0.001) (group difference in change p < 0.001) Pharmacist consultation decreased significantly in CHV only (− 24.1%, CI: − 37.9% to 10.4%; p = 0.001) and CHV +CCT (− 12.7%, CI: − 22.2% to 3.2%; p= 0.009) Decreased hospital visits among CHV only (−11.5%, CI: − 22.9% to 0.1%; p= 0.049) *Diabetes medication adherence and self-monitoring* Increase in adherence in the CHV + CCT group (6.8%, CI: 2.2 to 11.5%; p= 0.004) Decrease in self-monitoring CHV only participants (− 16.3%, CI: − 25.2% to 7.4%; p≤ 0.001) *Clinical measurements* Decrease in BMI in the CHV + CCT group (− 1.0 kg/m2, CI: − 1.7 to –0.3; p= 0.005) Decrease in HbA1C in CHV only 0.7% (CI: − 1.1% to 0.4%; p < 0.001), CHV +CCT − 0.5% (CI: − 0.7% to 0.3%; p < 0.001) and MPC group −0.2% (CI: −0.5 to 0.0%; p= 0.028) Increase in CHV+CCT group of normal blood pressure 11.3% (CI: 3.2 to 19.4%; p= 0.007)
Lyles *et al.* 2021 B^49^	Lebanon	Man-made crisis (refugees)	Multi-purpose unconditional cash transfer ~173.5 US$ per family per month (260 000 Lebanese pound) Time period not given.	Health service utilisation (care-seeking for children and chronic or acute illness among adults) Access to medication (Health expenditure)	*Health Service Utilisation (Care-seeking*) *For childhood illnesses*: increase was 19.3% (CI: 7.3,31.20%; p = 0.002) greater among MPC recipients compared to controls *For adult acute illnesses*: increased among MPC recipients but decreased in controls (adjusted diffference-in-difference (DiD) 11.3%; p = 0.057) *Childhood Hospitalisations:* significantly smaller among MPC recipients than among controls (adjusted difference − 6.1%, CI: − 11.7% to 0.4%; p = 0.037; effect size: −133.5%) *Access to medication* No significant changes observed within or between groups.
MacPherson and Sterck 2021^44^	Kenya	Man-made crises (refugee settlement)	Cash transfer (unclear whether conditional or unconditional) ~US$14–17 per person per month (unclear time period) (1400–1708 Kenyan Shilling)	DDS Calories per adult equivalent HFIAS Subjective well-being	*Diet and Nutrition* Refugees who received the transfer were found to have more diverse diets (20% higher DDS), higher caloric intake (p=0.12), and be less food secure (92% vs 79%) than those arriving just before. There was suggestive evidence that refugees living in Kalobeyei felt happier and more independent from aid than their counterparts in Kakuma. These results are robust to various tests and specification changes. kitchen-garden agriculture improves refugee diets *General Health and Wellbeing* Some evidence that the cash transfers had a positive effect on subjective well-being,. All coefficients were positive for effect on subjective well-being. In the non-parametric approach, the effect was statistically significant (p<0.01). However, with the parametric approach, coefficients were insignificant. The adjusted R^2^ of the parametric regression with predetermined variables was 0.01.
Moussa *et al.* 2022^50^	Lebanon	Man-made crises (refugees)	Multi-purpose unconditional cash transfer (monetary value unclear) Discontinued (12 months) Short run (up to 10 months) Long-term (16–22 months)	Acute illness Diarrhoea Respiratory infection Required primary healthcare Used primary healthcare	*Affected by Acute Illnesses* Lower likelihood of children 0–5 years reporting acute illnesses with MPCs Lower incidence of diarrhoea and respiratory infections in children 0–5 years with MPCs *Health Service Utilisation* Lower likelihood of needing PHC with MPCs More likely to use PHC when needed with MPCs *Sustainability of outcomes* Short-run improvement not sustainable when MPC benefits are discontinued, except for respiratory infections which don’t change Second cycle of cash transfer results in initial improvements of acute illnesses; needing PHC and using PHC maintain in the longer term
Schwab 2020^45^	Yemen	Man-made crises (human conflict)	Unconditional cash transfer ~US$147 over 3-month period in 3 monthly disbursements (10 500 Yemeni riyals)	HDSS FCS Value of food consumed Caloric intake	*Diet and Nutrition* Cash beneficiaries had a more diverse diet, fed infants and young children a wider variety of foods and consumed higher quality food. Cash beneficiaries also consumed approximately 150 less calories per day than food recipients. Self-reported measures of food insecurity incidents and non-food expenditures, including qat use, did not differ by transfer type.
Sibson *et al.* 2018^46^	Niger	Environmental (food crisis)	Unconditional cash transfer standard intervention = ~£144 over 4-month period in 4 monthly disbursements modified intervention = ~ £144 over 6-month period in 6 monthly disbursements	Acute malnutrition MUAC WHZ Dietary diversity	*Diet and Nutrition* There was no observable difference in the nutritional impact among children in the modified and standard cash transfer interventions. The odds of children having GAM and the adjusted mean WHZ were the same in each intervention arm and the general population. In children under 5, the GAM was 13.5% (95% CI: 10.8 to 16.8) at baseline and 14.7% (95% CI: 12.9 to 16.9, p=0.161) at endline. There was no significant difference in either the standard intervention (p=0.426) or the modified intervention (p=0.231).
Tossou *et al.* 2021^51^	Togo	Environmental (COVID-19 pandemic)	Unconditional cash transfer Monetary value and time period not given.	Healthcare utilisation	*Health Service Utilisation* For beneficiary households a positive impact of cash transfers on the use of healthcare services (66.6% higher in treatment group)

ANC, antenatal care; APAI, Acholi Psychosocial Assessment Instrument; CHV, community health volunteer; CHW, community healthcare worker; CTP, cash transfer programme; DDS, dietary diversity score; DRC, Democratic Republic of the Cong; FCS, food consumption score; HFIAS, household food insecurity access scale; HH, household; HHS, household hunger scale; IDP, internally displaced people; MAD, minimum acceptable diet; MDDW, minimum dietary diversity for women; MUAC, mid-upper arm circumference; PHQ-9, patient health questionnaire; PNC, postnatal care; PNCTP, Palestinian National Cash Transfer Programme; WG-SS, Washington group disability short set; WHZ, waist-to-hip ratio.

Cash transfers were implemented by governments (n=4)^29 35 43 51^ and humanitarian agencies/NGOs (n=19)^30–34 36–42 44–50^ such as the World Food Programme, United Nations Children’s Fund (UNICEF), UN High Commissioner for Refugees (UNHCR), and the International Rescue Committee (IRC). However, studies did not include sufficient detail describing the logistics/involvement regarding which actors served as programme implementers versus delivering cash. While only five studies reported specifically on health service utilisation,^35 48–51^ a range of health outcomes were investigated across the studies: nutrition-related outcomes (n=13),^30–34 36 39 42–47^ psychosocial and mental health (n=6),^29 31 36 38 40 41^ general/subjective self-reported health and well-being (n=3),^30 37^ acute illnesses (eg, diarrhoea, respiratory infection),^50^ diabetes control (eg, medication adherence, blood glucose self-monitoring, haemoglobin A1C (HbA1C) levels, Body Mass Index (BMI), blood pressure)^48^ or gender-based violence.^36^ Although not part of our inclusion criteria or our main outcome of interest, included studies also reported on expenditure on medicine/paying for healthcare (n=4)^29 30 40 49^ and self-reported access to health or medication (n=2).^41 49^ Analysis of qualitative data highlighted additional insight relating to mental health, access to health and the challenges relating to cash transfers (table 3).

**Table 3:** Qualitative data analysis

	Abu-Hamad *et al.* 2014^29^	Falb *et al.* 2020^36^	Freccero *et al.* 2019^37^	Gros *et al.* 2019^40^	Hagen-Zanker *et al.* 2018^41^
***Country***	Palestine	Syria	Afghanistan; Cameroon	Bangladesh	Jordan
***Humanitarian setting***	Man-made crises	Man-made crises	Man-made crises	Environmental	Man-made crises
**Mental health**					
Financial security	x	x	x		x
Personal security and autonomy			x		
Mental well-being		x		x	
**Access to Health**					
Cost of healthcare	x				x
Logistics and physical accessibility	x				x
**Challenges of cash transfers**					
Financial dependence and insecurity	x	x			
Exploitation and harm			x		
Logistic challenges			x		
Social stigma	x		x		
Sexual health			x		

### Quality assessment of individual studies

Though no article was excluded from the review synthesis based on quality, the results of the individual study quality appraisals are available in online supplemental table 3-6. Most studies demonstrated reasonable methodological quality, with quasiexperimental studies,^29 32 40 44 48 50^ indicating the highest quality assessment within the JBI checklist components for quasi-experimental studies. It was unclear whether strategies to address incomplete follow-up were utilised for any of the cohort studies,^31 33 34 36 49^ and confounders were either not clearly stated or were not included. The latter was similarly observed for the only cross-sectional study included.^51^ Two of the control trials^35 39^ were not randomised and, due to the nature of the intervention (receiving cash vs another type of assistance/no assistance), participants could not be blinded in the control trials.^30 35 38 39 42 43 45–47^ Furthermore, less than half of the qualitative studies located the researchers culturally and theoretically,^36 37^ and, therefore, did not address the role and influence of the researchers on their findings. It was also unclear for several qualitative studies whether obtained ethical approval.^40 41^

### Humanitarian setting characteristics

The included settings ranged across different types of humanitarian crises. Broadly, they can be divided into man-made disasters (n=17)^29 30 33–39 41 42 44 45 47–50^ and natural disasters (n=8).^31–34 40 43 46 51^ More specifically, these settings included (ongoing) conflict or civil war (n=10),^29 30 33–38 45 47^ refugee settings and internal displacement camps (n=7),^39 41 42 44 48–50^ drought (n=3),^33 34 43^ food crises (n=5),^31–34 46^ flooding (n=1),^40^ and the COVID-19 pandemic (n=1).^51^ Two studies reported on a combination of human conflict and drought in Somalia.^33 34^ The internal displacement camps were in Somalia^39^ and Ecuador,^42^ while refugee settlements based in Kenya hosted refugees escaping civil conflicts in Southern Sudan and Somalia.^44^ Both Jordan and Lebanon supported Syrian refugees escaping civil conflicts.^41 48–50^

### Cash transfer characteristics

The majority of the cash transfer programmes examined was unconditional (n=17).^29–31 33 34 36 38–42 45 46 48–51^ Although five studies included CCTs, one did not explicitly state the conditions to be met.^37 43 44 47 48^ The four other CCTs had different conditions: (1) for women to deliver their baby at a health facility,^35^ (2) attendance of sessions on child and infant feeding/care practises for mothers,^32^ (3) attendance of nutritional training sessions and compliance with child monitoring and treatment for malnutrition^47^ or (4) quarterly group education sessions on diabetes control, community health worker (CHW) home visits and the provision of receipts to prove use of appropriate services.^48^ For two studies, it was unclear whether the cash transfers were conditional or unconditional. Cash was usually provided in the short-term over several months (n=12)^30–34 36 37 39 42 45 46 50^ and ranged in amount; for example, US$96 total over 4 months,^46^ US$130 over 7 months,^30^ US$420 over 5 months,^39^ US$296 over 6 months.^31^ Fewer interventions utilised one-time cash transfers (n=3)^35 37 40^ or cash transfers that were provided for more than 12 months (n=2).^47 50^ The monetary value (n=5)^29 41 43 50 51^ and duration of cash transfer programmes (n=7)^29 41 43 44 48 49 51^ were not clearly provided in some studies. While all included cash transfers were used in humanitarian contexts, they varied in purpose: reducing (intergenerational) poverty and economic hardship (n=4)^29 38 43 50^; increasing households’ access to basic food and non-food needs (n=6)^30 37 41 44 48 49^; improving food security or preventing (child) acute malnutrition (including reducing child wasting or promote child weight gain) (n=9)^30–34 42 45–47^; preventing sale or loss of household assets (n=2),^31 40^ improvement of the use of maternal and child services (n=1)^35^; improving the ability to take preparatory early action ahead of a natural disaster (n=1)^40^; reducing the shocks caused by the COVID-19 pandemic^51^ and prevent negative impacts on health and livelihood (n=1).^40^ In two studies, the purpose of the cash transfer programme was not clearly defined.^36 39^

### General health and well-being

Only two studies reported on general health and well-being. Internally displaced persons in Cameroon (US$75–US$100 monthly over 5 months, US$43–US$84 monthly over 6 months) and Afghanistan (one-time US$80–US$198 over 2 months) reported general improvements in health, nutrition and housing after receiving multipurpose cash (MPC) transfers through an International Red Cross programme.^37^ Similarly, evidence from the Kalobeyei settlement in Kenya indicates that cash transfers (US$14 per person per month) positively impacted nutrition, subjective well-being and independence from aid.^44^ One study on UCTs (US$130 over 7 months) in the Democratic Republic of Congo presented contrasting evidence and suggested that prevalence of illness and deaths was similar between cash and voucher group.^30^

### Diet and nutrition-related outcomes

The most commonly investigated health outcomes were related to diets and nutrition.^31–34 36 39 42–47^ This was assessed in studies using a range of different metrics: the household hunger scale,^33^ dietary diversity (including, eg, minimum dietary diversity for women,^33 34 39 42 44^ household dietary diversity score (HDDS) and children’s dietary diversity),^31 32 43 45–47^ mid-upper arm circumference (MUAC),^31–34 39 46^ weight for height Z-score (WHZ),^31 32 39 46^ height for age Z-score,^47^ food consumption score (FCS),^39 42 44 45^ caloric intake,^42–45^ minimum acceptable diet,^34^ meal frequency,^31 33 34^ weight gain,^32^ weight gain velocity^32^ and the household food insecurity access scale.^36 44^ Six studies characterised the incidence and prevalence of acute malnutrition as measured by a WHZ < −2, an MUAC <125 mm or the presence of bilateral pitting oedema.^31–34 39 46^ The impact of cash transfers on nutrition varied. Different outcomes were reported based on the comparison group (eg, in-kind, voucher, no assistance), setting and programme. Studies presented both positive and null effects, rather than a consistently positive or negative effect as further described in the upcoming section.

Assessment of the impact of emergency CCTs (US$250 over 3 months) on the nutritional status of children in Niger found that the intervention was associated with a 1.27 kg overall weight gain (p value <0.001) and 1.82 greater increase in WHZ (p value <0.001) compared with the concurrent control group that did not receive the cash transfer. Furthermore, the odds of having acute malnutrition were 25 times higher for the comparison group.^32^ Previous evidence in the same setting indicated that among households targeted by emergency UCTs (US$296 over 6 months), diet-related factors and food expenditure for children were not associated with reduced risk of acute malnutrition.^31^ Likewise, evidence suggested that short-term emergency UCTs (monthly transfer of US$76 for 3 months) yielded significant improvements in food security in the Raqqa Governorate.^36^ In contrast, another monthly UCT (US$84 for 5 months) combined with a once-only distribution of a non-food-items kit and provision of piped water in Somalia found conflicting results when assessing its impact on acute malnutrition among children 6–59 months. Adjusted for age and sex, the intervention did not appear to reduce risk of acute malnutrition (HR 0.94, 95% CI 0.51 to 174) but did seem to increase the child dietary diversity score by 0.53 (95% CI 0.01 to 1.05).^39^ In Yemen, increases in dietary diversity (for both children and adults) were observed in the intervention group receiving CCT compared with the control group.^47^

Several studies compared the provision of cash transfers with other aid modalities. Two studies in Somalia assessed the impact of different emergency assistance modalities on acute malnutrition including in-kind food provision, food vouchers and UCTs (US$450 over 4 months).^33 34^Adjusted change in mean MUAC increased 0.1 cm (95% CI −0.1 to 0.4) in the mixed transfer (food, vouchers and unrestricted cash) recipients and 0.5 cm (95% CI 0.0 to 0.7) in the food voucher recipients. Adjusted prevalence of acute malnutrition in children under 5 decreased by 4.8% (95% CI −9.9 to 8.1) in mixed transfer recipients and increased by 0.7% (95% CI −13.4 to 14.4) compared with food voucher recipients. When comparing food voucher recipients with mixed transfer recipients, the change over time in both mean MUAC and prevalence of acute malnutrition was similar.^34^ Likewise, a UCT programme targeting pregnant and lactating women found no significant difference in preventing acute malnutrition compared with a control group with no cash-related intervention.^33^ When 6 monthly cash transfers (US$40 per month) were compared with food vouchers and food transfers in Northern Ecuador, all three arms significantly improved the quality and quantity of consumed food (measured by HDDS, Dietary Diversity Index (DDI), FCS, caloric intake, per capita food consumption). However, while the cash modality resulted in the most satisfaction among recipients (and food vouchers in the least), the increase in calories and dietary diversity were most cost-effectively accomplished by equal-valued food vouchers.^42^ In contrast, cash transfers in the Kalobeyei settlement were cheaper and more cost-effective than in-kind food assistance and were associated with better nutrition outcomes for refugees.^44^ Furthermore, a study in Yemen, a country with a study population similar to Niger with poor, rural households facing food insecurity, indicated that unconditional cash recipients (US$147 over 3 months) had more dietary diversity though they consumed 150 less calories a day per person than food recipients. Finally, when comparing a modified UCT (US$24 over 6 months) plus supplementary feeding with a standard UCT (US$36 over 4 months), the prevalence of acute malnutrition did not reduce (adjusted ORs (0.93 (95% CI (0.58 to 1.49), p=0.759) and 1.09 (95% CI (0.77, 1.55)), p=0.630) respectively), nor was the impact on food insecurity significantly different.^45^

### Diabetes control

One quasiexperimental study explored the impact of cash on diabetes control. The combined health education and CCT intervention programme (*condition:* quarterly group education sessions, home visits and provision of receipts for appropriate health services) was shown to be effective in improving diabetes control (demonstrated by lower HbA1C and improved diabetes medication adherence), blood pressure control and reductions in BMI among Syrian refugees with Type II diabetes. Notably, the education intervention alone was effective in improving diabetes control, while an unconditional MPC transfer alone was less effective.^48^

### Psychosocial and mental health

The second most commonly examined health outcome related to psychosocial and mental health. These findings were often self-reported or measured by validated tools/questionnaires (eg, the patient health questionnaire). While psychosocial and mental health were not always the intended targets of cash transfer programmes, multiple studies reported on these effects.

A study in Raqqa Governorate, grappling with a dual crisis from the Islamic State of Iraq and Syrian occupation and civil conflict, found that an UCT (US$228 over 3 months) implemented by the IRC resulted in no change in perceived serious household needs and daily stressors (β=0.12; 95% CI −0.24 to 0.48) and an increase in depressive symptoms (β=0.89; 95% CI 0.34 to 1.43) before and after cash distribution.^36^ Additionally, a study in Northern Uganda that combined cash transfers with business skills training indicated that there was no significant alleviation in depression.^38^ Poor communication about logistics and timing of cash transfers ending, caused stress and anxiety among participants and their relationships.^36 37^

Despite this, several studies reported positive impacts of cash transfers on mental health (n=4), despite this not being the intent of the cash transfer. Several women in the Raqqa Governorate reported in qualitative interviews that their levels of stress, as well as feelings of humiliation and shame, were reduced in the period of cash assistance delivery. ‘*The cash we received maintained our dignity and met our needs. I don’t live like a queen because of the aid, but it is good’,* mentioned a divorced woman living with her in-laws.^36^ Similarly, a study in Bangladesh indicated that forecast-based UCTs (US$60 one-time) reduced psychosocial stress during and after the flood when compared with a group that did not receive cash.^40^ An UNHCR UCT programme in Jordan for Syrian refugees reduced stress and anxiety among beneficiaries. One third of the respondents in the study indicated their mental well-being had improved and that stress related to inability to pay their rent was alleviated. One of the male beneficiaries reported that, ‘*receiving the UNHCR cash transfer changed our life on all moral and financial aspects, I no longer worry about rent and it eased pressure on the entire family’*.^41^ This was consistent with earlier findings from the Palestinian National Cash Transfer Programme on Gazan children.^29^

### Acute childhood illnesses

A singular study explored the impact of cash on acute illnesses, comparing discontinued recipient households, short-term recipient households, long-term recipient households and non-beneficiary households of MPC transfers. In children under five, short-term and long-term participants suffered less acute illnesses than non-recipients. This finding was further confirmed with lower incidence of specific acute diseases such as diarrhoea and respiratory infections in recipient children versus non-recipient children.^50^

### Health service utilisation, access to health and health expenditure

The five studies on health service utilisation in this review focused on overall healthcare utilisation,^51^ maternal and newborn care,^35^ needed and accessed primary healthcare (PHC),^50^ diabetes service utilisation,^48^ care-seeking behaviour for children and chronic or acute illness among adults.^49^ While almost all positive, there were mixed results relating to what degree CCTs improved healthcare utilisation.

Two studies reported on how CCTs affected maternal and child healthcare utilisation. Mothers who received US$15 if they delivered a child at a health facility in Afghanistan reported an increase in both maternal and newborn service usage and CHW home visits. However, only the increase in antenatal care (ANC) visits was statistically significant (adjusted mean difference 45% (95% CI 0.18 to 0.72), *p* value 0.004). In contrast, the mean difference in postnatal care (PNC) visits, CHW home visits and facility deliverywere not statistically significant.^35^ Furthermore, MPC recipients (~173.5 US$ per family per month in Lebanon) reported a 19.3% (CI: 7.3,31.20%; p = 0.002) greater increase in care-seeking behaviour for childhood illnesses compared with controls, and a significantly smaller increase in child hospitalisations among MPC recipients than controls (DiD −6.1%; p = 0.037).^49^ Likewise, a lower likelihood for needing PHC and a higher likelihood of seeking PHC when needed was observed among children under 5 years old from MPC recipient families.^50^

A study conducted in a population of Syrian refugees compared the effects of CCTs alone, health education alone and CCTs plus health education on healthcare utilisation for diabetes.^48^ The study found that the CCT plus health education group had the most significant increases in monthly medication spending (13.6%, p < 0.001) and outpatient diabetes visits (25.3%, p< 0.001). Additionally, the health education only group experienced a decreased overall spending on diabetes care (− 18.7%, p= 0.001).^48^ This study overall concluded that CCTs were most effective when combined with the health education intervention.

A study on survey data in Togo showed that a government CCT programme, the NOVISSI scheme, improved healthcare utilisation during the economic hardship from COVID-19.^51^ When recipients of the Togo CCT were matched based on demographics to non-recipients, they were 66% more likely to access healthcare and less likely to use traditional medicine.^51^ While no other studies in this review reported on health service utilisation, some had secondary findings that suggested various effects. A study evaluating UNHCR UCTs in Jordan indicated that the transfers enabled some beneficiaries to partially cover the costs of treatment. While this may alleviate some of the costs of accessing healthcare, it was not decisive in accessing healthcare.^52^ This same issue was reported by a respondent in Palestine who stated ‘*We still don’t have money for medicines, so we use the cash to pay off debts at the pharmacy’.*^29^ This was further reflected in Bangladesh where, even though forecast-based UCTs were used the second most frequently on health expenses following food, it did not result in significant differences in the experience of illness compared with the comparison group (intervention 17.8% of 152; comparison 20.1% of 149, p value 0.60).^40^

### Enabling and constraining factors to cash transfer implementation

Only a few studies reported on enabling or constraining factors around cash transfer implementation. Enabling factors mentioned were lower costs for implementing agencies compared with other modalities (eg, vouchers),^30 33 34^ giving households the freedom of choice,^30^ enabling policy environments^41^ and beneficiaries’ preference for cash.^34^ Yet, several factors may constrain successful implementation including: weak health service infrastructure,^31^ reduced onsite access,^35^ dysfunctional markets or limited market choices,^39^ beneficiary decision-making (especially of caregivers),^31^ barriers to accessing cash programming by specific groups because of, for example, their gender or age,^37^ struggle to access cash due to travel limitations^46^ or a lack of concrete risk assessment tools that prevent meaningful consultation with community members into the programme design.^37^ Additionally, logistical challenges such as physical and institutional barriers to access, methods of disbursement and corruption or bribery were mentioned.^37^

## Discussion

This review presents evidence on the impact of cash on health in humanitarian settings. Most studies were on UCTs in human conflict or food crisis settings discussing their impact on nutrition, psychosocial and mental health or general health and well-being. While the purpose of several programmes was specifically aimed at improving food security and preventing malnutrition, few were specifically designed with as purpose to address other health outcomes.^30–34 36 39 42–47^ Only five studies reported on the utilisation of health services.^35 48–51^ Nineteen studies reported some positive impacts on various health outcomes and use of health services,^29 31 33–38 40–45 47–51^ 11 reported no statistically significant impact on outcomes assessed^30 35 36 38–40 44 46–49^ and 4 reported potential negative impacts on health outcomes.^36 37 42 45^ While the majority of studies described nutrition and diet-related outcomes, the impact cash transfers had on these health outcomes varied. Although, most studies reported decreased frequency of acute malnutrition or related metrics (eg, dietary diversity) in cash groups compared with in-kind food, food voucher and/or no assistance, two studies found that cash beneficiaries consumed less calories than in-kind food beneficiaries.^42 45^ Furthermore, while most studies reported positive impacts on psychosocial and mental health, often related to alleviating the stress of financial burdens, two studies reported social exclusion of recipients and verbal abuse from non-beneficieries.^36 37^

Our findings are broadly in line with evidence from research on cash transfers’ impact on health outside of humanitarian settings. For example, a 2010 Cochrane review on CCTs in low and middle-income countries (LMICs) reported that despite methodological weaknesses, the evidence suggests that cash transfers may contribute to health benefits.^10^ Likewise, systematic reviews published 3 and 5 years later, respectively, on maternal, child and newborn health and CCTs suggested that cash transfers are effective in addressing child health determinants (eg, access to healthcare, morbidity risk),^24^ and that CCTs improved ANC visits, delivery at a health facility, skilled attendance at birth, reduction of low birth weight incidence and increased tetanus toxoid vaccination of mothers.^24^ When exploring the wider (grey) literature from a humanitarian perspective, a Humanitarian Policy Group 2012 report indicated that there is some evidence backing up the use of cash transfers to improve nutritional status in emergency settings if markets are functioning and quality food is available.^53^ Finally, a 2015 Cochrane review on cash transfers in humanitarian disasters in LMICs concluded that studies either reported improved outcomes or no statistically significant evidence of UCTs impacting health outcomes. However, they considered the body of evidence to be of very low quality with great uncertainty across all outcomes.^22^ The findings of this review are compatible with these previous reviews: there is evidence to suggest a positive relationship between cash transfers and health outcomes; however, there is a need for stronger additional high-quality evidence that can also be synthesised through a meta-analysis to determine the extent of this impact. Additionally, there may be unintended consequences from cash transfers that should be further explored.

The included studies were heterogeneous in their approaches to examine the effect of cash. Some studies investigated the impact of cash compared with food vouchers or in-kind food, while others used a comparison group that did not receive any assistance, tested the value/distribution method of cash transfers or performed pre–post implementation comparisons. Testing the implementation of cash transfers in one group against a group with no aid assistance also posed ethical challenges. Consequently, it is difficult to ascribe the extent or magnitude of the effect due to cash transfers versus other mechanisms. Studies comparing cash to vouchers, for example, often reported there was not a statistically significant difference in their impact. However, there is often a preference for cash transfers over other aid forms.^13 14^ With this in mind, policymakers and programme designers should consider the risks and benefits of these different approaches before implementing these interventions.

Included were limited in the time frames; studies were conducted over a few months (2 months) to a few years (18–24 months). Additionally, the length of cash transfers varied, from one-time disbursements to monthly allotments over a 7-month period. Going forward, it will be important to measure outcomes on a larger scale over a longer period of time, to fully understand whether cash can offer sustainable, long-lasting positive health impacts. Future research and aid provision could also consider the length of time covered by the cash transfers themselves and the effect of distributing the same monetary value over shorter versus longer time periods. The health outcomes examined across the studies were also fairly limited in scope, with only one study exploring the impact of cash on acute illnesses. This could be due to the requirement of laboratory-intensive or invasive measurements for some health outcomes in order to ascertain disease/health. Yet, cash transfers may also positively contribute to a range of other communicable (eg, COVID-19, Ebola) and non-communicable diseases. Several cash transfer programmes have been brought in place in the response to COVID-19 over 2020–2021 globally. This includes the expansion of the two largest existing cash transfer programmes in Colombia (Families in Action and Youth in Action) by lowering eligibility thresholds and including education and mental and psychosocial health targets.^54 55^ However, while several studies on cash transfers during COVID-19 were retrieved in our search strategy, the majority did not focus on the cash transfers’ impact on human health but rather on mitigating the economic impacts of the pandemic.^56–59^

Interestingly, the vast majority of cash transfers included were not specifically designed to cover health expenses. This has two important implications. First, there could be potential bias induced when cash is distributed for a specific purpose and communicated as such—resulting in recipients aiming to conform to what they were told the cash should be spent on (eg, food). Second, if the cash transfer value has not been designated to cover health expenses, households may likely trade-off and prioritise different expenses including health. As cash transfers become increasingly common, it will be important for implementers to collect, analyse and share the data on the effectiveness of their interventions in order to inform future programmes and evidence-based. Documenting best practices and considerations on safe and ethical implementation are important considerations. Therefore, it may be useful for future studies to adapt a similar framework or investigate a consistent group of core metrics in order to assuage some of the heterogeneity of this literature base. Additionally, future research and documentation of evidence could consider the roles of the different actors involved in the conceptualisation, development and delivery of cash transfers. It is important to consider how different implementers may have different motives and do not necessarily obey the same humanitarian principles, which can expose beneficiaries to different risks.^60^

The type of data collected and reported is also an important consideration; qualitative data may provide further detail and insight into the experiences and perspectives of recipients and implementers. The qualitative data synthesised here highlighted some of the possible unintended consequences or impacts such as social exclusion, community tension and verbal abuse.^36 37^ Humanitarian settings pose additional challenges that must be considered, in terms of collecting data and adapting the intervention in real time where appropriate.^61^ Innovative social science and anthropology methodologies in implementation research that emphasise the influence of context, and unique experiences of different populations and settings may prove useful in investigations of cash transfers in humanitarian settings.^53 62^ These approaches do this by providing not only a positivist or binary outcome about the effectiveness of programmes but also more experiential insight into what works and does not work.

One of the limitations of this study is that it focuses on cash and does not include vouchers, which have been increasingly used to improve accessibility to health facilities. Second, we focused on the direct impact of cash transfers on health outcomes and service utilisation without exploring wider social determinants indirectly affecting health, such poverty reduction, clean water and sanitation access and education. Third, the available evidence was limited, and studies often had significant limitations, complicating robust information synthesis and preventing the performance of additional analysis (eg, meta-analyses). Yet, opportunities for rigorous approaches in acute emergencies are limited due to inaccessibility and the short planning cycles of intervention design and implementation. Finally, we focused on the inclusion of peer-reviewed academic journals in order to limit potential biases and confounders, inaccuracies and incomplete information and to ensure the replicability of this review.^63^ Consequently, evidence reported in the grey literature (including NGO and government reports, theses and dissertations) that provide data not found in the peer-reviewed published academic literature may have been missed, resulting in a chance of publication bias as authors tend to publish studies with significant results. Despite this, the review has several strengths, including a detailed and updated comprehensive search strategy to gather available evidence, the synthesis of both quantitative and qualitative literature, unlimited date range of publications included and a broad definition of health outcomes allowing for a diverse examination of the impact of cash transfers on health. To our knowledge, this is the first systematic review on both conditional and unconditional cash transfers and health outcomes in humanitarian settings. The range of data presented in this review emphasises that the impact of cash transfers is not homogeneous across settings due to differences in exposure and nature of disasters, vulnerability, sensitivity and adaptive capacity of the population in a humanitarian setting. For example, both the transfer value and timeliness of distribution are components of cash transfers that influence their effectiveness.

The findings from this systematic review exhibit not only the potential impact of cash transfers on health outcomes and health service utilisation but also calls for future research. There is urgent need for high-quality quantitative and qualitative empirical evidence that is methodologically robust, investigates a range of humanitarian settings, and is conducted over longer time periods to better understand the long-term impacts of cash transfers on health and health service utilisation in humanitarian settings. Future research must investigate this area in further detail to better understand the specific variables that influence the effectiveness of cash transfers on health outcomes. For example, considering the types of crises (armed conflict vs epidemics) or health metrics (chronic vs infectious diseases). These lines of investigation could also provide insight to the impact of cash transfers on health outcomes beyond nutrition and mental/psychosocial health that were most examined in this review. Additionally, there is a need for further and clearer evidence on implementation factors that shape how cash transfers may function in a setting. For example, across health and international development interventions, it is highly encouraged and even expected to involve stakeholders directly at the beginning of a programme rather than to introduce an intervention from the outside or top-down approaches. Humanitarian or emergency settings may pose unique challenges when it comes to the timeliness and logistics of response and so future work may consider the role of building local capacity within cash transfer and other programme that can be leveraged in times of need. The findings of this review, as well as this call for further research, can have implications for both policy and practice by informing the development of evidence-based cash transfer programmes as they are implemented across humanitarian settings.

## Data Availability

All data relevant to the study are included in the article or uploaded as supplementary information. Not applicable.
