# Supplementary material for: Impact of conditional and unconditional cash transfers on health outcomes and use of health services in humanitarian settings: a mixed-methods systematic review
Source: BMJ Glob Health. 2022 Jan 25;7(1):e007902. doi: 10.1136/bmjgh-2021-007902 (PMC8796230; doi:10.1136/bmjgh-2021-007902)
Supplement: Supplementary data [file bmjgh-2021-007902supp001.pdf]

Supplement Materials

The impact of conditional and unconditional cash transfers on health outcomes and use of health services in humanitarian settings: a mixed-methods systematic review

Supplement table 1 Search Strategy used on 24th February 2021 and re-run the 8th December 2021

<table>
<tr><th>Database</th><th>Terms</th></tr>
<tr><td>Medline via Ovid

2,801 results</td><td>1 (cash transfer* or income supplement* or (mone* adj3 (incentiv* or grant* or transfer*)) or (social adj (welfare* or assistance*)) or voucher* or food stamp* or mobile money or e-cash* or e-transfer* or e-wallet* or cash* or CCT or coupon* or direct payment* or ((conditional or unconditional or restricted or unrestricted) adj3 transfer*)).ti,ab. or Social Welfare/ or exp food assistance/ 33559
2 (Genocide* or war or warfare or (armed adj2 (intervention or conflict)) or conflict affected or displaced or displacement or refugee* or asylum* or migra* or (emergency adj2 (response* or relief*)) or humanitarian or disaster* or (relief adj2 (plan* or Work* or effort*)) or mass casualty or disaster* or hurricane* or typhoon* or storm* or tornado* or blizzard* or flood* or drought* or cyclone* or rain* or wind* or heatwave* or cold wave* or fire* or wildfire* or tsunami* or avalanche* or landslide* or rockslide* or mudslide* or tidal wave* or typhoon* or volcanic eruption* or volcan* or earthquake* or famine* or starv* or food insecurity or homeless* or housing insecurity or (outbreak* and disease*) or epidemic* or pandemic* or ((crop* or agriculture) adj3 (fail* or devast*))).ti,ab. or (relief work/ or exp mass casualty incident/ or exp armed conflicts/ or refugees/ or exp extreme weather/ or exp natural disasters/ or exp disease outbreaks/ or exp food security/ or exp food insecurity/ or exp genocide/ or exp warfare/ or exp homeless persons/ or exp famine/) 1215745
3 1 and 2 2801</td></tr>
<tr><td>PubMed

2,671 results</td><td>(("Relief Work"[Mesh:NoExp] OR "Mass Casualty Incidents"[MeSH Terms] OR "Armed Conflicts"[MeSH Terms] OR "Refugees"[MeSH Terms] OR "Extreme Weather"[MeSH Terms] OR "natural disasters"[MeSH Terms] OR "disease outbreaks" [MeSH Terms] OR "food security" [MeSH Terms] OR "humanitarian emergency"[Title/Abstract] OR "genocide*"[Title/Abstract] OR "war"[Title/Abstract] OR "armed intervention"[Title/Abstract] OR "armed conflict"[Title/Abstract] OR "conflict affected"[Title/Abstract] OR "displaced*"[Title/Abstract] OR "displacement"[Title/Abstract] OR "refugee*"[Title/Abstract] OR "asylum"[Title/Abstract] OR "migrat*"[Title/Abstract] OR "emergency response*"[Title/Abstract] OR "emergency relief"[Title/Abstract] OR "humanitarian"[Title/Abstract] OR "disaster*"[Title/Abstract] OR "relief planning"[Title/Abstract] OR "Relief Work"[Title/Abstract] OR "Relief effort"[Title/Abstract] OR "mass casualty"[Title/Abstract] OR "disaster*"[Title/Abstract] OR "hurricane*"[Title/Abstract] OR "typhoon*"[Title/Abstract] OR "storm*"[Title/Abstract] OR "tornado*"[Title/Abstract] OR "blizzard*"[Title/Abstract] OR "flood*"[Title/Abstract] OR "drought*"[Title/Abstract] OR "cyclone*"[Title/Abstract] OR "rain*"[Title/Abstract] OR "wind*"[Title/Abstract] OR "heatwave*"[Title/Abstract] OR "cold wave*"[Title/Abstract] OR "fire*"[Title/Abstract] OR "wildfire*"[Title/Abstract] OR "tsunami*"[Title/Abstract] OR "avalanche*"[Title/Abstract] OR "landslide*"[Title/Abstract] OR "rockslide*"[Title/Abstract] OR "mudslide*"[Title/Abstract] OR "tidal wave"[Title/Abstract] OR "typhoon*"[Title/Abstract] OR "volcanic eruption*"[Title/Abstract] OR "volcan*"[Title/Abstract] OR "earthquake*"[Title/Abstract] OR "famine*"[Title/Abstract] OR "starv*"[Title/Abstract] OR "food insecurity"[Title/Abstract] OR "homeless*"[Title/Abstract] OR "housing insecurity"[Title/Abstract] OR (outbreak*[Title/Abstract] AND disease*[Title/Abstract]) OR "epidemic*"[Title/Abstract] OR "pandemic*"[Title/Abstract] OR ((crop[Title/Abstract] OR agriculture[Title/Abstract]) AND (failure[Title/Abstract] OR fail[Title/Abstract] OR devastate[Title/Abstract] OR devastation[Title/Abstract]))))
AND
(("cash transfer"[Title/Abstract] OR "income supplement*"[Title/Abstract] OR "monetary incentive"[Title/Abstract] OR "monetary grant"[Title/Abstract] OR "social welfare*"[Title/Abstract] OR "social assistance*"[Title/Abstract] OR "voucher*"[Title/Abstract] OR "commodity voucher*"[Title/Abstract] OR "value voucher*"[Title/Abstract] OR "food stamp*"[Title/Abstract] OR "mobile money"[Title/Abstract] OR "e-cash*"[Title/Abstract] OR "e-transfer*"[Title/Abstract] OR "e-wallet*"[Title/Abstract] OR "cash*"[Title/Abstract] OR "CCT"[Title/Abstract] OR "coupon*"[Title/Abstract] OR "direct payment"[Title/Abstract] OR "money transfer"[Title/Abstract] OR "conditional transfer*"[Title/Abstract] OR "unconditional transfer*"[Title/Abstract] OR "restricted transfer*"[Title/Abstract] OR "unrestricted transfer*"[Title/Abstract] OR ("monetary transfer"[Title/Abstract]) OR "Social Welfare"[Mesh:NoExp] OR "food assistance"[MeSH Terms]))</td></tr>
<tr><td>EMBASE via Ovid

2,484 results</td><td>1 (cash transfer* or income supplement* or (mone* adj3 (incentiv* or grant* or transfer*)) or (social adj (welfare* or assistance*)) or voucher* or food stamp* or mobile money or e-cash* or e-transfer* or e-wallet* or cash* or CCT or coupon* or direct payment* or ((conditional or unconditional or restricted or unrestricted) adj3 transfer*)).ti,ab. or exp *Social Welfare/ or exp *food assistance/ 36078
2 (Genocide* or war or warfare or (armed adj2 (intervention or conflict*)) or conflict affected or displaced or displacement or refugee* or asylum* or migra* or (emergency adj2 (response* or relief*)) or humanitarian or disaster* or (relief adj2 (plan* or Work* or effort*)) or mass casualty or disaster* or hurricane* or typhoon* or storm* or tornado* or blizzard* or flood* or drought* or cyclone* or rain* or wind* or heatwave* or cold wave* or fire* or wildfire* or tsunami* or avalanche* or landslide* or rockslide* or mudslide* or tidal wave* or typhoon* or volcanic eruption* or volcan* or earthquake* or famine* or starv* or food insecurity or homeless* or housing insecurity or (outbreak* and disease*) or epidemic* or pandemic* or ((crop* or agriculture) adj3 (fail* or devast*))).ti,ab. or (*relief work/ or exp *disaster/ or exp *mass disaster/ or exp *refugee/ or exp *extreme weather/ or exp *natural disaster/ or exp *epidemic/ or exp *food security/ or exp *food insecurity/ or exp *genocide/ or exp *war/ or exp *homeless person/ or exp *hunger/) 1347530
3 1 and 2 2484</td></tr>
<tr><td>CINAHL via EbscoHost

1,792 results</td><td><table>
<tr><th>#</th><th>Query</th><th>Results</th></tr>
<tr><td>S7</td><td>S5 AND S6</td><td>1,792</td></tr>
<tr><td>S6</td><td>S3 OR S4</td><td>328,395</td></tr>
<tr><td>S5</td><td>S1 OR S2</td><td>18,303</td></tr>
<tr><td>S4</td><td>(MH "War+") OR (MH "Refugees+") OR (MH "Humanitarian Aid") OR (MH "Disaster Planning") OR (MH "Disasters+") OR (MH "Mass Casualty Incidents") OR (MH "Natural Disasters+") OR (MH "Weather+") OR (MH "Extreme Weather") OR (MH "Disease Outbreaks+") OR (MH "Homeless Persons") OR (MH "Homelessness")</td><td>131,694</td></tr>
<tr><td>S3</td><td>(Genocide* or war or warfare or (armed N2 (intervention or conflict)) or “conflict affected” or displaced or displacement or refugee* or asylum* or migra* or (emergency n2 (response* or relief*)) or humanitarian or disaster* or (relief n2 (plan* or Work* or effort*)) or “mass casualty” or disaster* or hurricane* or typhoon* or storm* or tornado* or blizzard* or flood* or drought* or cyclone* or</td><td>300,924</td></tr>
</table></td></tr>
</table>

		rain* or wind* or heatwave* or cold wave* or fire* or wildfire* or tsunami* or avalanche* or landslide* or rockslide* or mudslide* or tidal wave* or typhoon* or "volcanic eruption*" or volcan* or earthquake* or famine* or starv* or "food insecurity" or homeless* or "housing insecurity" or (outbreak* n5 disease*) or epidemic* or pandemic* or ((crop* or agriculture) n3 (fail* or devast*)))	
	S2	(MH "Food Assistance") OR (MH "Social Welfare")	7,904
	S1	("cash transfer*" or "income supplement*" or (mone* n3 (incentiv* or grant* or transfer*)) or (social n1 (welfare* or assistance*)) or voucher* or "food stamp*" or "mobile money" or e-cash* or e-transfer* or e-wallet* or cash* or CCT or coupon* or "direct payment*" or ((conditional or unconditional or restricted or unrestricted) n3 transfer*))	17,016

Global Health via EbscoHost

1,843 results

#	Query	Results
S8	S6 AND S7	1,843
S7	S2 OR S5	242,081
S6	S1 OR S4	11,605
S5	(((((((DE "war") OR (DE "displacement")) OR (DE "refugees"))) OR (DE "disasters" OR DE "natural disasters" OR DE "agricultural disasters")) OR (DE "weather" OR DE "fire weather")) OR (DE "natural disasters")) OR (DE "food security")) OR (DE "homeless people")	26,985
S4	((DE "social welfare" OR DE "child welfare" OR DE "rural welfare")) OR (DE "Food Stamp Program")	2,305
S3	S1 AND S2	1,564
S2	(Genocide* or war or warfare or (armed N2 (intervention or conflict)) or "conflict affected" or displaced or displacement or refugee* or asylum* or migra* or (emergency n2 (response* or relief*)) or humanitarian or disaster* or (relief n2 (plan* or Work* or effort*)) or "mass casualty" or disaster* or hurricane* or typhoon* or storm* or tornado* or blizzard* or flood* or drought* or cyclone* or rain* or wind* or heatwave* or cold wave* or fire* or wildfire* or tsunami* or avalanche* or landslide* or rockslide* or mudslide* or tidal wave* or typhoon* or "volcanic eruption*" or volcan* or earthquake* or famine* or starv* or "food insecurity" or homeless* or "housing insecurity" or (outbreak* n5 disease*) or epidemic* or pandemic* or ((crop* or agriculture) n3 (fail* or devast*)))	234,625
S1	("cash transfer*" or "income supplement*" or (mone* n3 (incentiv* or grant* or transfer*)) or (social n1 (welfare* or assistance*)) or voucher* or "food stamp*" or "mobile money" or e-cash* or e-transfer* or e-wallet* or cash* or CCT or coupon* or "direct payment*" or ((conditional or unconditional or restricted or unrestricted) n3 transfer*))	11,259

Scopus

9,312 results

TITLE-ABS("cash transfer*" or "income supplement*" or (mone* w/3 (incentiv* or grant* or transfer*)) or (social w/1 (welfare* or assistance*)) or voucher* or "food stamp*" or "mobile money" or e-cash* or e-transfer* or e-wallet* or cash* or CCT or coupon* or "direct payment*" or ((conditional or unconditional or restricted or unrestricted) w/3 transfer*)) and TITLE-ABS(Genocide* or war or warfare or (armed w/2 (intervention or conflict)) or "conflict affected" or displaced or displacement or refugee* or asylum* or migra* or (emergency w/2 (response* or relief*)) or humanitarian or disaster* or (relief w/2 (plan* or Work* or effort*)) or "mass casualty" or disaster* or hurricane* or typhoon* or storm* or tornado* or blizzard* or flood* or drought* or cyclone* or rain* or wind* or heatwave* or "cold wave*" or fire* or wildfire* or tsunami* or avalanche* or landslide* or rockslide* or mudslide* or "tidal wave*" or typhoon* or "volcanic eruption*" or volcan* or earthquake* or famine* or starv* or "food insecurity" or homeless* or "housing insecurity" or (outbreak* w/5 disease*) or epidemic* or pandemic* or ((crop* or agriculture) w/3 (fail* or devast*)))

Web of Science Core Collection

5,751 results

#	Query	Results
# 3	#2 AND #1<br>Indexes=SCI-EXPANDED, SSCI, A&HCI Timespan=All years	5,751
# 2	TS=(Genocide* or war or warfare or (armed near/2 (intervention or conflict)) or "conflict affected" or displaced or displacement or refugee* or asylum* or migra* or (emergency near/2 (response* or relief*)) or humanitarian or disaster* or (relief near/2 (plan* or Work* or effort*)) or "mass casualty" or disaster* or hurricane* or typhoon* or storm* or tornado* or blizzard* or flood* or drought* or cyclone* or rain* or wind* or heatwave* or cold wave* or fire* or wildfire* or tsunami* or avalanche* or landslide* or rockslide* or mudslide* or tidal wave* or typhoon* or "volcanic eruption*" or volcan* or earthquake* or famine* or starv* or "food insecurity" or homeless* or "housing insecurity" or (outbreak* near/5 disease*) or epidemic* or pandemic* or ((crop* or agriculture) near/3 (fail* or devast*)))<br>Indexes=SCIELO Timespan=All years	50,411

	# 1	TS=(“cash transfer*” or “income supplement*” or (mone* near/3 (incentiv* or grant* or transfer*)) or (social near/1 (welfare* or assistance*)) or voucher* or “food stamp*” or “mobile money” or e-cash* or e-transfer* or e-wallet* or cash* or CCT or coupon* or “direct payment*” or ((conditional or unconditional or restricted or unrestricted) near/3 transfer*))<br>Indexes=SCIELO Timespan=All years	3,118
**SciELO via Web of Science**<br><br>249 results	**#**	**Query**	**Results**
	# 3	#2 AND #1<br>Indexes=SCIELO Timespan=All years	249
	# 2	TS=(Genocide* or war or warfare or (armed near/2 (intervention or conflict affected” or displaced or displacement or refugee* or asylum* or migra* or (emergency near/2 (response* or relief*)) or humanitarian or disaster* or (relief near/2 (plan* or Work* or effort*)) or “mass casualty” or disaster* or hurricane* or typhoon* or storm* or tornado* or blizzard or flood* or drought* or cyclone* or rain* or wind* or heatwave* or cold wave* or fire* or wildfire* or tsunami* or avalanche* or landslide* or rockslide* or mudslide* or tidal wave* or typhoon* or “volcan ic eruption*” or volcan* or earthquake* or famine* or starv* or “food insecurity” or homeless* or “housing insecurity” or (outbreak* near/5 disease*) or epidemic* or pandemic* or ((crop* or agriculture) near/3 (fail* or devast*)))<br>Indexes=SCI-EXPANDED, SSCI, A&HCI Timespan=All years	2,994,269
	# 1	TS=(“cash transfer*” or “income supplement*” or (mone* near/3 (incentiv* or grant* or transfer*)) or (social near/1 (welfare* or assistance*)) or voucher* or “food stamp*” or “mobile money” or e-cash* or e-transfer* or e-wallet* or cash* or CCT or coupon* or “direct payment*” or ((conditional or unconditional or restricted or unrestricted) near/3 transfer*))<br>Indexes=SCI-EXPANDED, SSCI, A&HCI Timespan=All years	75,461
**LILACS**<br><br>7 results	cash transfer" or coupon or voucher or "social welfare" or "mobile money" or "direct payment" [Words] and disaster or war or humanitarian or famine or refugee or storm or "armed conflict" [Words]		

Supplement table 2 of included cohort studies (n=4) using the Joanna Briggs Institute critical appraisal tool.[8]

Study	Were the two groups similar and recruited from the same population?	Were the exposures measured similarly to assign people to both exposed and unexposed groups?	Was the exposure measured in a valid and reliable way?	Were confounding factors identified?	Were strategies to deal with confounding factors stated?	Were the groups/participants free of the outcome at the start of the study (or at the moment of exposure)?	Were the outcomes measured in a valid and reliable way?	Was the follow up time reported and sufficient to be long enough for outcomes to occur?	Was follow up complete, and if not, were the reasons to loss to follow up described and explored?	Were strategies to address incomplete follow up utilized?	Was appropriate statistical analysis used?
Bliss *et al.* 2016[31]	yes	yes	yes	no	no	yes	yes	no	yes	unclear	yes
Doocy *et al.* 2020 A[33]	yes	yes	yes	unclear	unclear	no	yes	yes	yes	unclear	yes
Doocy *et al.* 2020 B[34]	yes	yes	yes	unclear	unclear	no	yes	yes	yes	unclear	yes
Falb *et al.* 2020[36]	yes	yes	yes	yes	yes	yes	yes	no	yes	unclear	yes
Lyles *et al*. 2021 B[49]	yes	yes	yes	Unclear	unclear	NA	yes	yes	yes	unclear	yes

Supplement table 3 of included qualitative studies (n=4) using the Joanna Briggs Institute critical appraisal tool.[8]

Study	Is there congruity between the stated philosophical perspective and the research methodology?	Is there congruity between the research methodology and the research question or objectives?	Is there congruity between the research methodology and the methods used to collect data?	Is there congruity between the research methodology and the representation and analysis of data?	Is there congruity between the research methodology and the interpretation of results?	Is there a statement locating the researcher culturally or theoretically ?	Is the influence of the researcher on the research, and vice- versa, addressed?	Are participants, and their voices, adequately represented?	Is the research ethical according to current criteria or, for recent studies, and is there evidence of ethical approval by an appropriate body?	Were strategies to address incomplete follow up utilized?
Abu-Hamad *et al.* 2014[29]	yes	yes	yes	yes	yes	no	unclear	yes	yes	not applicable
Falb *et al.* 2020[36]	yes	yes	yes	yes	yes	yes	yes	yes	yes	not applicable
Freccero *et al.* 2019[37]	unclear	yes	unclear	yes	yes	yes	yes	yes	yes	not applicable
Gros *et al.* 2019[40]	yes	yes	yes	yes	yes	no	no	yes	no	not applicable
Hagen-Zanker *et al.* 2018[52]	unclear	yes	unclear	unclear	unclear	unclear	yes	unclear	unclear	not applicable

Supplement table 4 of included quasi-experimental studies (n=5) using the Joanna Briggs Institute critical appraisal tool.[8]

Study	Is it clear in the study what is the 'cause' and what is the 'effect' (i.e. there is no confusion about which variable comes first)?	Were the participants included in any comparisons similar?	Were the participants included in any comparisons receiving similar treatment/care, other than the exposure or intervention of interest?	Was there a control group?	Were there multiple measurements of the outcome both pre- and post-the intervention/exposure?	Was follow up complete and if not, were differences between groups in terms of their follow up adequately described and analysed?	Were the outcomes of participants included in any comparisons measured in the same way?	Were outcomes measured in a reliable way?	Was appropriate statistical analysis used?
Abu-Hamad *et al.* 2014[29]	yes	yes	yes	yes	yes	unclear	yes	yes	yes
Bliss *et al.* 2018[32]	yes	yes	yes	yes	yes	yes	yes	unclear	yes
Gros *et al.* 2019[40]	yes	yes	yes	yes	no	yes	yes	yes	yes
MacPherson *et al.* 2021[44]	yes	yes	yes	no	yes	yes	yes	yes	yes
Lyles *et al.* 2021 A[48]	yes	yes	yes	yes	yes	yes	yes	yes	yes
Moussa *et al.* 2021[50]	yes	yes	yes	yes	no	no	yes	yes	

Supplement table 5 of included randomised control trials (n=8) using the Joanna Briggs Institute critical appraisal tool.[8]

Study	Was true randomization used for assignment of participants to treatment groups?	Was allocation to treatment groups concealed?	Were treatment groups similar at the baseline?	Were participants blind to treatment assignment?	Were those delivering treatment blind to treatment assignment?	Were outcomes assessors blind to treatment assignment?	Were treatment groups treated identically other than the intervention of interest?	Was follow up complete and if not, were differences between groups in terms of their follow up adequately described and analysed?	Were participants analysed in the groups to which they were randomized?	Were outcomes measured in the same way for treatment groups?	Were outcomes measured in a reliable way?	Was appropriate statistical analysis used?	Was the trial design appropriate, and any deviations from the standard RCT design (individual randomization, parallel groups) accounted for in the conduct and analysis of the trial?
Aker *et al.* 2017[30]	yes	no	yes	no	no	unclear	yes	yes	yes	yes	yes	yes	no
Edmond *et al.* 2019[35] *	not applicable	no	yes	no	yes	yes	yes	yes	yes	yes	yes	yes	yes
Green *et al.* 2016[38]	yes	no	yes	no	no	no	yes	yes	yes	yes	yes	yes	yes
Grijalva-Eternod *et al.* 2018[39] **	not applicable	no	yes	no	no	no	yes	yes	not applicable	yes	yes	yes	yes
Hidrobo *et al.* 2014[42]	yes	yes	yes	no	no	no	yes	yes	yes	yes	yes	yes	yes
Hou *et al.* 2010[43]	yes	yes	yes	no	no	no	yes	yes	yes	yes	yes	yes	yes
Schwab *et al.* 2019[45]	yes	no	yes	no	no	no	yes	yes	yes	yes	yes	yes	yes
Sibson *et al.* 2018[46]	no	unclear	yes	no	no	no	unclear	unclear	yes	yes	yes	yes	yes

Kurdi *et al.* 2021[47]	no	unclear	yes	no	no	unclear	yes	no	yes/no	yes	Yes	yes	yes

*Non-randomised population-based intervention study **Non-randomised cluster trial

Supplement table 6 of included cross-sectional studies (n=1) using the Joanna Briggs Institute critical appraisal tool.[8]

Study	Were the criteria for inclusion in the sample clearly defined?	Were the study subjects and the setting described in detail?	Was the exposure measured in a valid and reliable way?	Were objective, standard criteria used for measurement of the condition?	Were confounding factors identified?	Were strategies to deal with confounding factors stated?	Were the outcomes measured in a valid and reliable way?	Was appropriate statistical analysis used?
Tossou 2021[51]	yes	no	yes	unclear	unclear	unclear	unclear	yes/no

Supplement table 7 Preferred Items for Systematic Reviews and Met-analyses (PRISMA) Checklist.[7]

Section and Topic	Item #	Checklist item	Location where item is reported
**TITLE**			
Title	1	Identify the report as a systematic review.	Page 1
**ABSTRACT**			
Abstract	2	See the PRISMA 2020 for Abstracts checklist.	Page 2
**INTRODUCTION**			
Rationale	3	Describe the rationale for the review in the context of existing knowledge.	Page 4-5
Objectives	4	Provide an explicit statement of the objective(s) or question(s) the review addresses.	Page-4-5
**METHODS**			
Eligibility criteria	5	Specify the inclusion and exclusion criteria for the review and how studies were grouped for the syntheses.	Page 6
Information sources	6	Specify all databases, registers, websites, organisations, reference lists and other sources searched or consulted to identify studies. Specify the date when each source was last searched or consulted.	Page 6
Search strategy	7	Present the full search strategies for all databases, registers and websites, including any filters and limits used.	Page 5-7, Supplement table 7
Selection process	8	Specify the methods used to decide whether a study met the inclusion criteria of the review, including how many reviewers screened each record and each report retrieved, whether they worked independently, and if applicable, details of automation tools used in the process.	Page 5-7
Data collection process	9	Specify the methods used to collect data from reports, including how many reviewers collected data from each report, whether they worked independently, any processes for obtaining or confirming data from study investigators, and if applicable, details of automation tools used in the process.	Page 5-7
Data items	10a	List and define all outcomes for which data were sought. Specify whether all results that were compatible with each outcome domain in each study were sought (e.g. for all measures, time points, analyses), and if not, the methods used to decide which results to collect.	Page 5-6
	10b	List and define all other variables for which data were sought (e.g. participant and intervention characteristics, funding sources). Describe any assumptions made about any missing or unclear information.	Page 7
Study risk of bias assessment	11	Specify the methods used to assess risk of bias in the included studies, including details of the tool(s) used, how many reviewers assessed each study and whether they worked independently, and if applicable, details of automation tools used in the process.	Page 7
Effect measures	12	Specify for each outcome the effect measure(s) (e.g. risk ratio, mean difference) used in the synthesis or presentation of results.	Page 6-7
Synthesis methods	13a	Describe the processes used to decide which studies were eligible for each synthesis (e.g. tabulating the study intervention characteristics and comparing against the planned groups for each synthesis (item #5)).	Page 6-7
	13b	Describe any methods required to prepare the data for presentation or synthesis, such as handling of missing summary statistics, or data conversions.	Page 7

	13c	Describe any methods used to tabulate or visually display results of individual studies and syntheses.	Page 7
	13d	Describe any methods used to synthesize results and provide a rationale for the choice(s). If meta-analysis was performed, describe the model(s), method(s) to identify the presence and extent of statistical heterogeneity, and software package(s) used.	Page 7
	13e	Describe any methods used to explore possible causes of heterogeneity among study results (e.g. subgroup analysis, meta-regression).	Page 7
	13f	Describe any sensitivity analyses conducted to assess robustness of the synthesized results.	NA
Reporting bias assessment	14	Describe any methods used to assess risk of bias due to missing results in a synthesis (arising from reporting biases).	Page 7
Certainty assessment	15	Describe any methods used to assess certainty (or confidence) in the body of evidence for an outcome.	Page 7
**RESULTS**			
Study selection	16a	Describe the results of the search and selection process, from the number of records identified in the search to the number of studies included in the review, ideally using a flow diagram.	Page 7-8, Figure 1
	16b	Cite studies that might appear to meet the inclusion criteria, but which were excluded, and explain why they were excluded.	NA
Study characteristics	17	Cite each included study and present its characteristics.	Page 7-8
Risk of bias in studies	18	Present assessments of risk of bias for each included study.	Page 8
Results of individual studies	19	For all outcomes, present, for each study: (a) summary statistics for each group (where appropriate) and (b) an effect estimate and its precision (e.g. confidence/credible interval), ideally using structured tables or plots.	Page 7-13 Tables 1-3
Results of syntheses	20a	For each synthesis, briefly summarise the characteristics and risk of bias among contributing studies.	Page 7-13
	20b	Present results of all statistical syntheses conducted. If meta-analysis was done, present for each the summary estimate and its precision (e.g. confidence/credible interval) and measures of statistical heterogeneity. If comparing groups, describe the direction of the effect.	NA
	20c	Present results of all investigations of possible causes of heterogeneity among study results.	NA
	20d	Present results of all sensitivity analyses conducted to assess the robustness of the synthesized results.	NA
Reporting biases	21	Present assessments of risk of bias due to missing results (arising from reporting biases) for each synthesis assessed.	NA
Certainty of evidence	22	Present assessments of certainty (or confidence) in the body of evidence for each outcome assessed.	Page 7-13
**DISCUSSION**			
Discussion	23a	Provide a general interpretation of the results in the context of other evidence.	Page 13-14
	23b	Discuss any limitations of the evidence included in the review.	Page 13-18
	23c	Discuss any limitations of the review processes used.	Page 16-17

	23d	Discuss implications of the results for practice, policy, and future research.	Page 17-18
**OTHER INFORMATION**			
Registration and protocol	24a	Provide registration information for the review, including register name and registration number, or state that the review was not registered.	Page 5
	24b	Indicate where the review protocol can be accessed, or state that a protocol was not prepared.	Page 5
	24c	Describe and explain any amendments to information provided at registration or in the protocol.	Page 5
Support	25	Describe sources of financial or non-financial support for the review, and the role of the funders or sponsors in the review.	Page 18
Competing interests	26	Declare any competing interests of review authors.	Page 18
Availability of data, code and other materials	27	Report which of the following are publicly available and where they can be found: template data collection forms; data extracted from included studies; data used for all analyses; analytic code; any other materials used in the review.	Page 18
